# Phenotypic Profiling Reveals that *Candida albicans* Opaque Cells Represent a Metabolically Specialized Cell State Compared to Default White Cells

**DOI:** 10.1128/mBio.01269-16

**Published:** 2016-11-22

**Authors:** Iuliana V. Ene, Matthew B. Lohse, Adrian V. Vladu, Joachim Morschhäuser, Alexander D. Johnson, Richard J. Bennett

**Affiliations:** a Department of Molecular Microbiology and Immunology, Brown University, Providence, Rhode Island, USA; b Department of Microbiology, University of California, San Francisco, San Francisco, California, USA; c Department of Mathematics, MIT, Cambridge, Massachusetts, USA; d Institut für Molekulare Infektionsbiologie, Universität Würzburg, Würzburg, Germany; e Department of Biochemistry and Biophysics, University of California, San Francisco, San Francisco, California, USA

## Abstract

The white-opaque switch is a bistable, epigenetic transition affecting multiple traits in *Candida albicans* including mating, immunogenicity, and niche specificity. To compare how the two cell states respond to external cues, we examined the fitness, phenotypic switching, and filamentation properties of white cells and opaque cells under 1,440 different conditions at 25°C and 37°C. We demonstrate that white and opaque cells display striking differences in their integration of metabolic and thermal cues, so that the two states exhibit optimal fitness under distinct conditions. White cells were fitter than opaque cells under a wide range of environmental conditions, including growth at various pHs and in the presence of chemical stresses or antifungal drugs. This difference was exacerbated at 37°C, consistent with white cells being the default state of *C. albicans* in the mammalian host. In contrast, opaque cells showed greater fitness than white cells under select nutritional conditions, including growth on diverse peptides at 25°C. We further demonstrate that filamentation is significantly rewired between the two states, with white and opaque cells undergoing filamentous growth in response to distinct external cues. Genetic analysis was used to identify signaling pathways impacting the white-opaque transition both *in vitro* and in a murine model of commensal colonization, and three sugar sensing pathways are revealed as regulators of the switch. Together, these findings establish that white and opaque cells are programmed for differential integration of metabolic and thermal cues and that opaque cells represent a more metabolically specialized cell state than the default white state.

## IMPORTANCE

Epigenetic transitions are an important mechanism by which microbes adapt to external stimuli. For *Candida albicans*, such transitions are crucial for adaptation to complex, fluctuating environments, and therefore contribute to its success as a human pathogen. The white-opaque switch modulates multiple *C. albicans* attributes, from sexual competency to niche specificity. Here, we demonstrate that metabolic circuits are extensively rewired between white and opaque states, so that the two cell types exhibit optimal fitness under different nutritional conditions and at different temperatures. We thereby establish that epigenetic events can profoundly alter the metabolism of fungal cells. We also demonstrate that epigenetic switching regulates filamentation and biofilm formation, two phenotypes closely associated with pathogenesis. These experiments reveal that white cells, considered the most clinically relevant form of *C. albicans*, are a “general-purpose” state suited to many environments, whereas opaque cells appear to represent a more metabolically specialized form of the species.

## INTRODUCTION

Many cells can undergo epigenetic, heritable transitions without changes in the primary DNA sequence, and such transitions are a key source of heterogeneity in the microbial world (1–3). This type of heterogeneity is an effective strategy for microbes to deal with dynamic environments, where alternative cell states may be optimized for different conditions. Such “bet hedging” has been shown to benefit cells exposed to stressful conditions, as demonstrated for subpopulations of *Saccharomyces cerevisiae* cells during heat stress (4). Phenotypic variation has also been linked to metabolic flexibility, as it enables rapid population growth even after large-scale changes in available nutrients (5–8). While the advantages of bet hedging do not require interactions between different cell states, population heterogeneity can also support a division of labor whereby distinct individual organisms cooperate for the greater good. A prominent example is found in *Bacillus subtilis*, where different cell subtypes cooperate to support complex multicellular communities (9–11).

*Candida albicans* is a prevalent fungal pathogen that shares a dynamic relationship with its human host. *C. albicans* exists as a commensal species in humans, colonizing multiple mucosal surfaces of the body, but it is also an opportunistic pathogen capable of causing life-threatening systemic infections (12). This species exhibits extensive phenotypic plasticity; it can grow as single-celled yeast or multicellular hyphae, and can undergo epigenetic switching between alternative cell states. It also demonstrates considerable metabolic flexibility, which is important for adaptation to diverse host niches and for virulence (13–20).

Epigenetic switching in *C. albicans* is best exemplified by the white-opaque switch, in which cells undergo heritable and reversible switching between two morphologically distinct cell types (21). White and opaque cells exhibit a number of contrasting properties, including differences in mating competency, phagocytosis by host cells, virulence, and niche specificity (22–27). Switching between white and opaque states is usually seen in *MTL* homozygous strains and occurs stochastically at low frequency under standard laboratory conditions (26). Opaque cells are generally unstable at mammalian body temperature (37ºC) and undergo mass conversion to the white state (26). However, environmental stimuli such as *N*-acetylglucosamine (GlcNAc), nutrient limitation, anaerobic conditions, and carbon dioxide all modulate switching frequencies between the two cell types and can promote opaque cell stability at high temperatures (24, 28–33). Transcriptional profiling has revealed extensive differences in the expression of metabolic genes between white and opaque cells (34, 35). In particular, tricarboxylic acid (TCA) cycle and fatty acid β-oxidation genes are upregulated in opaque cells, whereas glycolytic genes are upregulated in white cells (34, 35). Despite these differences, a systematic analysis of the metabolic preferences of white and opaque cells has not been performed, nor has there been a detailed examination of the impact of external cues on important phenotypes.

The transcriptional regulation of the white-opaque switch in *C. albicans* has been dissected in depth and involves overlapping feedback loops between eight transcription factors (Wor1, Wor2, Wor3, Wor4, Efg1, Czf1, Ahr1, and Ssn6) (36–44). White cell formation is promoted by Efg1 (38, 40, 44, 45), which is also a transcriptional regulator of filamentation and carbon metabolism (46, 47). Deletion of Efg1 affects both commensalism and virulence in animal models of infection, underlying its central role in mediating interactions between *C. albicans* and the host (48–52). The master regulator of the opaque state is Wor1, whose expression is both necessary and sufficient to drive switching to opaque (41–43). Wor1 expression also impacts fitness in the host; ectopic *WOR1* overexpression can promote formation of the “GUT” state, in which cells are optimized for colonization of the gastrointestinal tract (52). Genetic analyses have established close mechanistic links between white-opaque switching and other developmental programs such as filamentation, yet it is unclear how these programs are coregulated or how they are integrated with metabolic cues.

In this study, we perform a high-throughput analysis of *C. albicans* white cells and opaque cells grown on 1,440 different metabolic and chemical substrates at two different temperatures. We sought to answer the following questions. (i) How does the fitness of white and opaque cells compare on different nutrients? (ii) How do environmental cues impact other phenotypes such as white-opaque switching and filamentation? (iii) What signaling pathways influence nutrient-induced white-opaque switching? (iv) Finally, how do thermal cues impact each of these traits? Our experiments establish that white and opaque states are wired differently for interactions with the environment—they are programmed to undergo optimal growth, filamentation, and biofilm formation on different nutrients and at different temperatures.

## RESULTS

### Global analysis of the metabolic profiles of *C. albicans* white and opaque cells.

**FIG 1:**
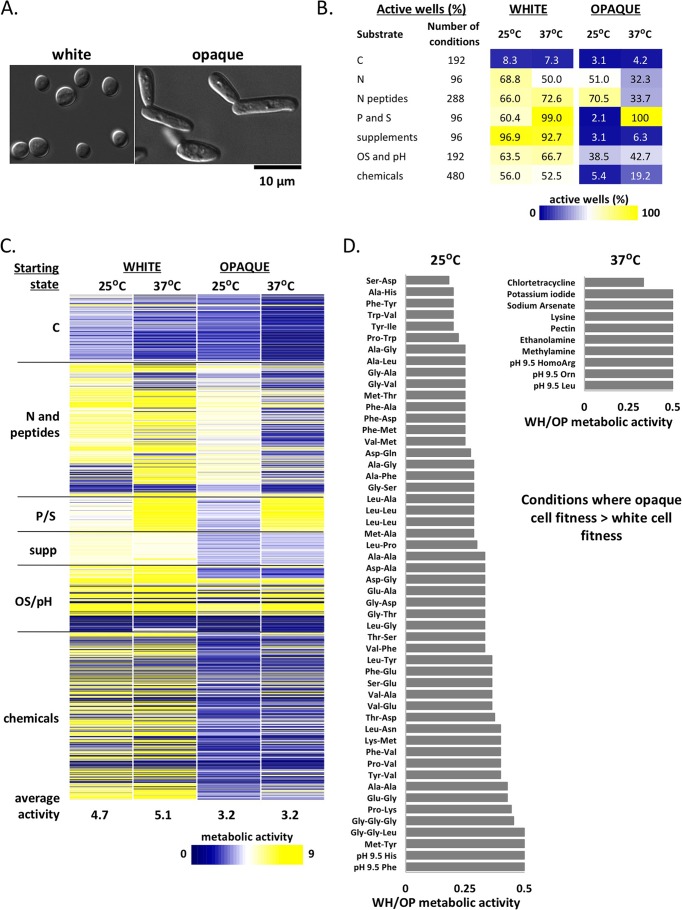
Metabolic profiling of wild-type white and opaque cells. (A) Cell morphologies of white and opaque cells. (B) Percent active wells (metabolic activity of ≥5) for each substrate group for white and opaque cells at 25°C and 37°C. (C) Heat map of metabolic activities of white and opaque cells grown at 25°C and 37°C in Phenotypic MicroArray (PM) conditions. Metabolic activity is represented on a scale from 0 (no growth [blue]) to 9 (maximum growth [yellow]). Substrate groups are C sources (PM01 and PM02), N and peptide sources (PM03 and PM06 to PM08), P and S sources (P/S) (PM04), supplements (supp) (PM05), osmotic stress and pH substrates (OS/pH) (PM09 and PM10), and chemicals (PM21 to PM25). Average metabolic activities across all conditions for each starting state are shown at the bottom of the heat map. (D) Conditions in which opaque cells grow better than white cells based on white cell/opaque cell (WH/OP) metabolic activity ratios that were ≤0.5.

Phenotypic MicroArrays provide a high-throughput tool for examining microbial cells under thousands of culture conditions. To assess phenotypic differences between *C. albicans* white (WH) and opaque (OP) cells (Fig. 1A), we grew both cell types on Phenotypic MicroArray (PM) plates (Biolog) coated with different nutrients and chemical substrates. Growth of white and opaque cells was monitored at 25°C and 37°C for a period of 24 to 48 h. Growth data were analyzed using DuctApe, which weighs cell fitness by combining multiple growth parameters (length of the lag phase, slope of the growth curve, average height of the curve, maximum cell respiration, and area under the curve; see Materials and Methods; also see data in Table S1A in the supplemental material). At the end of the growth period, cells were also analyzed to ascertain whether the induction of filamentous growth or switching between white and opaque forms took place during the experiment (see Materials and Methods).

We compared growth of the two cell types on different carbon sources (PM plates PM01 and PM02), nitrogen sources (PM03), phosphorus-sulfur sources (PM04), nutritional supplements (PM05; e.g., folic acid, biotin), nitrogen peptides (PM06 to PM08), osmotic stress and pH (PM09 and PM10), as well as chemical agents (PM21 to PM25; e.g., antifungals, antimicrobials, chelators, toxic ions). Analysis of 1,440 PM wells both at 25°C and at 37°C revealed that white cells generally grew better than opaque cells (Fig. 1B and C; see Table S1A in the supplemental material). Of the 2,880 evaluated conditions, the white cell/opaque cell (WH/OP) metabolic ratio was >2 in 855 conditions, whereas this ratio was <0.5 in only 62 conditions, indicating that white cells are fitter than opaque cells under many *in vitro* culture conditions.

We noted that *C. albicans* growth was particularly sensitive to changes in the carbon (C) source, as both cell types grew poorly when cultured on a number of alternative C sources. Thus, only 8% of C source wells showed “active” growth (DuctApe activity index of 5 or greater) when white cells were cultured at 25°C or 37°C, compared to >50% of wells in other PM plates where glucose was present as the C source (Fig. 1B). Opaque cells had a similar dependence on C source, with less than 4% of wells containing different C sources showing “active” growth (Fig. 1B).

Next, we considered the effect of temperature on the relative fitness of white and opaque cells. White cells were, on average, 1.5-fold more active than opaque cells at both temperatures, and this difference was exacerbated at 37°C (Fig. 1C, see average metabolic activities). Conditions in which white cells had metabolic activities greater than those of opaque cells were common across all PM plates. For example, the WH/OP metabolic ratio was ≥5 when cells were grown on carbohydrates such as fucose and xylitol, on dipeptides enriched for methionine, or on numerous stress conditions at both temperatures (see Table S1A in the supplemental material). Conditions in which opaque cells displayed higher metabolic activities than white cells (WH/OP metabolic ratio of <0.5) were also identified at both temperatures, although these were more frequent at 25°C (52 conditions at 25°C versus 10 conditions at 37°C [Fig. 1D]). These conditions included growth on diverse dipeptides and tripeptides enriched for alanine and glycine (including Gly-Gly-Gly) at 25°C, as well as growth at pH 9.5 at both temperatures (Fig. 1D).

To more closely examine global fitness differences between white and opaque cells, we determined the ratio of their metabolic activities across different PM categories (e.g., C sources). The average ratio of white cell/opaque cell metabolic activities was >1 for all categories at both 25°C and 37°C (see Fig. S1A in the supplemental material), indicating that white cells have a general fitness advantage over opaque cells under most nutrient conditions and at both temperatures. This difference was greatest (>2-fold) for C sources or nitrogen (N) peptides at 37°C, as well as for various “chemicals” at both temperatures (Fig. S1A). Growth in the presence of chemicals/antimicrobials (PM21 to PM25) revealed that, with a few exceptions, white cells displayed a significant advantage in withstanding stressful conditions (Fig. S2G and Table S1A). This trend was also seen in the presence of antifungals—white cells had equal or higher metabolic activities relative to opaque cells at both 25°C and 37°C (Fig. S2H). Detailed analyses for white and opaque cells grown on different nutrients and chemicals are provided in Text S1 and Fig. S1.

These observations establish that the fitness of both cell types is critically dependent on nutritional and thermal cues. White and opaque cell types were particularly sensitive to changes in C source, and these differences were exacerbated by temperature. Furthermore, white cells were as fit, if not fitter, than opaque cells under the majority of tested conditions. This establishes that the two cell types have distinct metabolic properties, with direct implications for the apparent prevalence of white cells during host infections.

### Thermal regulation of white and opaque cell fitness.

**FIG 2:**
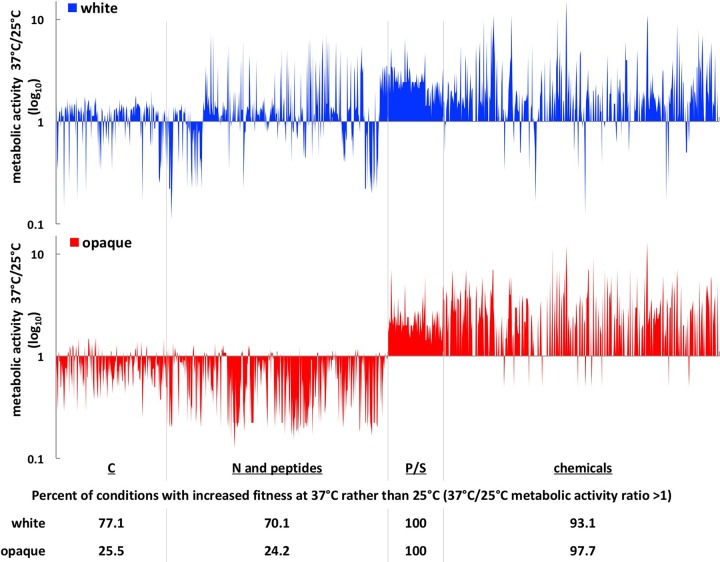
Impact of temperature on cell type fitness across PM conditions. The metabolic activities of white and opaque cells grown at 25°C and 37°C for 24 h were compared for each PM condition. Ratios of 37°C/25°C metabolic activities are shown for C, N and peptide, P/S, and chemical substrates, on a log_10_ scale. The percentage of wells with increased fitness at 37°C rather than 25°C (ratio of 37°C/25°C metabolic activity of >1) is shown below each category.

To further understand the relationship between temperature, nutritional cues, and cell fitness, we more closely compared the metabolic activities of white and opaque cells at 25°C and 37°C on different nutrients. When grown on different C sources, white cells generally displayed increased fitness at 37°C compared to 25°C, with 77% of conditions showing a 37°C/25°C metabolic activity ratio of >1 (Fig. 2). In contrast, opaque cells showed a 37°C/25°C ratio of >1 for only 25.5% of these wells, indicating that opaque cells are fitter at ambient temperature rather than body temperature on most C substrates. Exceptions to these trends included growth on several sugars (including maltose, sucrose, ribose, and galactose), where both cell types were fitter at 37°C than at 25°C (7% of C sources). Conversely, growth on many amino acids or certain carboxylic acids (14% of C sources, including malic, oxalic, and butyric acids) resulted in greater fitness for both cell types at 25°C compared to 37°C (see Fig. S1I and Table S1B in the supplemental material).

Similar temperature dependencies were observed when examining growth of white and opaque cells on different N and peptide substrates (PM03 and PM06 to PM08 [Fig. 2]). Thus, white cells displayed increased metabolic activities at 37°C relative to 25°C when grown on 70% of N sources and N peptides. In contrast, opaque cells were more metabolically active at 25°C than at 37°C for 75% of these substrates (Fig. 2; see Fig. S1J in the supplemental material). Again, a subset of conditions (15%) promoted more efficient growth of both cell types at 25°C than at 37°C, but very few conditions (0.8%) promoted more efficient growth of both cell types at 37°C than at 25°C (Table S1B).

**FIG 3:**
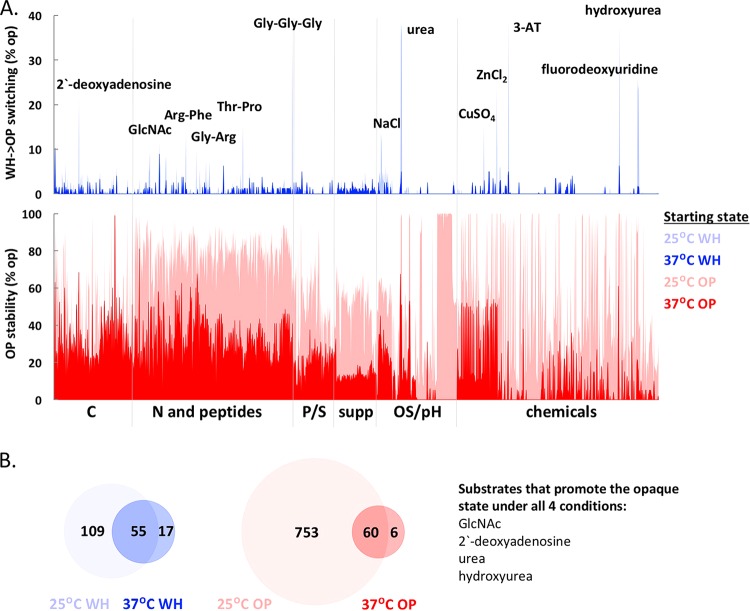
White-opaque switching is regulated by metabolic cues and by temperature. (A) (Top) White-to-opaque switching rates at 25°C and 37°C on different substrates. Individual substrates that induced the highest levels of white-to-opaque switching are indicated. (Bottom) Opaque cell stability at 25°C and 37°C on different substrates. (B) Overlap between conditions that induce >2% switching in white cells at 25°C and 37°C and between conditions that stabilize the opaque state (>50% opaque cells at the end of the experiment) at 25°C and 37°C. Four substrates induced white-to-opaque switching and stabilized the opaque state at both temperatures.

Both cell types generally displayed increased fitness at 37°C than at 25°C when grown on phosphorus and sulfur sources (P/S) or when challenged with various “chemicals” (Fig. 2). This may reflect the high levels of opaque-to-white switching occurring at 37°C under these conditions (Fig. 3A, discussed below), as well as the increased fitness of white cells at 37°C. Overall, our results reveal the following: (i) the metabolism of white and opaque cells is biased for optimal growth at different temperatures, with white cells generally fitter at 37°C than at 25°C, whereas the opposite is true for opaque cells; (ii) specific nutrients determine the effect of temperature on growth, with some nutrients supporting efficient growth at one temperature over the other independent of the phenotypic state of the cell.

### Effects of environmental cues on white-opaque switching.

A notable property of the white-opaque switch is its high sensitivity to multiple environmental cues. Opaque cells have been observed to switch *en masse* to the white state when cultured at 37°C, whereas white-to-opaque switching is induced by multiple stimuli including *N*-acetylglucosamine (GlcNAc), CO_2_, low oxygen, and genotoxic or oxidative stress (25, 30–32). Both white-to-opaque (WH→OP) and opaque-to-white (OP→WH) switching frequencies were monitored by visual analysis of cell morphologies in our assays (see Table S1C in the supplemental material), as well as by culturing cells on agar plates following completion of the PM plate assays.

### (i) White-to-opaque switching.

Only 8% of the ~2,900 culture conditions induced >2% white-to-opaque switching, with most switching observed at 25°C rather than at 37°C (Fig. 3A; see Fig. S2A and S2B in the supplemental material). Certain C sources, N peptides, and several “chemicals” induced the highest switching frequencies in a temperature-dependent manner (Fig. 3A). The strongest inducers of white-to-opaque switching included 2′-deoxyadenosine, GlcNAc variants, dipeptides and tripeptides containing alanine, glycine, or arginine (e.g., Ala-Arg, Gly-Arg, Gly-Pro, or Gly-Gly-Gly), high NaCl concentrations (5 to 6%), fluorodeoxyuridine, hydroxyurea, 3-amino-1,2,4-triazole (3-AT) and several metal salts (Fig. 3A; Table S1D). Several alternative C sources also induced low levels (2 to 3%) of white-to-opaque switching at 37°C. Unlike the majority of the PM plates, these conditions lacked glucose, suggesting that the absence of glucose might promote stable white-to-opaque switching at this temperature, as discussed below.

### (ii) Opaque cell stability.

Opaque cells are often unstable when cultured at 37°C, switching to the white state *en masse* (21, 53). In agreement with previous studies, we observed that the majority of conditions at 37°C led to opaque cells switching efficiently to the white state (Fig. 3A; see Fig. S2C in the supplemental material). Comparison of switching frequencies at the two temperatures showed that 92% of the PM conditions where opaque cells were stable (defined as wells where >50% cells retained the opaque state) were at 25°C (Fig. 3A and Fig. S2A and S2D). Conditions that promoted opaque cell stability at 25°C included different N and peptide sources, growth at pH 9.5, and several carboxylic acids (including butyric, succinic, capric, caproic, and malic acids) where >90% of cells retained the opaque state at the end of the experiment (Fig. 3A; Table S1D).

At 37°C, 37% of PM conditions induced opaque-to-white switching *en masse* (defined as >90% of the population in the white state at the end of the experiment). These conditions included most of the chemicals, osmotic stress, and pH substrates (see Fig. S2C and Table S1D in the supplemental material). Only 5% of the PM conditions at 37°C promoted opaque cell stability, and almost half of these (32 out of 66 [Fig. S2A]) showed no detectable growth, suggesting that active cell division is required for phenotypic switching, in agreement with previous results (53, 54). The other conditions supporting stable opaque cell propagation at 37°C included dipeptides containing alanine or glycine and alternative carbon sources such as sorbitol or GlcNAc variants (Table S1D). Conditions that stabilized the opaque state at 37°C also induced the white-to-opaque switch at 25°C (e.g., GlcNAc both stabilized the opaque state and promoted white-to-opaque switching) (Fig. 3B).

We also evaluated whether environmental cues similarly impact white-opaque switching frequencies in strain backgrounds other than *C. albicans* SC5314. To test this, we performed switching assays under a subset of conditions using three additional *C. albicans* strains (WO-1, P37005 and L26) that are homozygous at the *MTL* locus and therefore competent for white-opaque switching. In general, we observed that different strain backgrounds responded in a qualitatively similar manner to external cues, although the amplitude of the observed effects varied considerably between strains (see Table S1I in the supplemental material). For example, the WO-1 strain consistently displayed higher rates of white-to-opaque switching relative to the other three strains. Thus, in the presence of glucose, 14% of WO-1 white cells switched to the opaque state at 25°C, whereas no switching (<0.5%) was detected in the other strain backgrounds. White-to-opaque switching frequencies increased in all four strains in the presence of 1% GlcNAc, but WO-1 again showed a higher switching rate (58%) relative to the other three strains (2 to 5% switching [Table S1I]). In addition, combining several inducing cues resulted in increased rates of white-to-opaque switching, so that a mixture of GlcNAc−2′-deoxyadenosine−Gly-Gly-Gly−urea−NaCl (each at 1%) resulted in 36% of SC5314-derived white cells switching to the opaque state, whereas any one of these chemicals alone produced less than 6% switching in this strain background (Table S1I).

These experiments provide a global analysis of the environmental cues that impact white-opaque switching. In addition to known switching cues (e.g., elevated temperature, GlcNAc, and hydroxyurea), we uncovered multiple novel cues, including a number of diverse dipeptides and chemical substrates (see Fig. S3A and B in the supplemental material). Interestingly, several of the environmental cues that showed the strongest impact on switching frequencies also influenced other phenotypic traits like filamentation, as discussed in the next section.

### Filamentation of white and opaque cells in response to environmental stimuli.

The yeast-hyphal switch plays a key role in the pathogenicity of *C. albicans* (55–57). Filamentous growth was previously shown to be induced by distinct environmental cues in white and opaque cells, as filamentation in white cells was favored at 37°C and by cues such as peptidoglycan (58), whereas opaque cells underwent efficient filamentation at 25°C in response to different nutritional cues (59). To examine this difference in more detail, we tested each of the 1,440 culture conditions for their ability to induce white or opaque cell filamentation at both 25°C and 37°C.

### (i) Filamentation of white cells.

**FIG 4:**
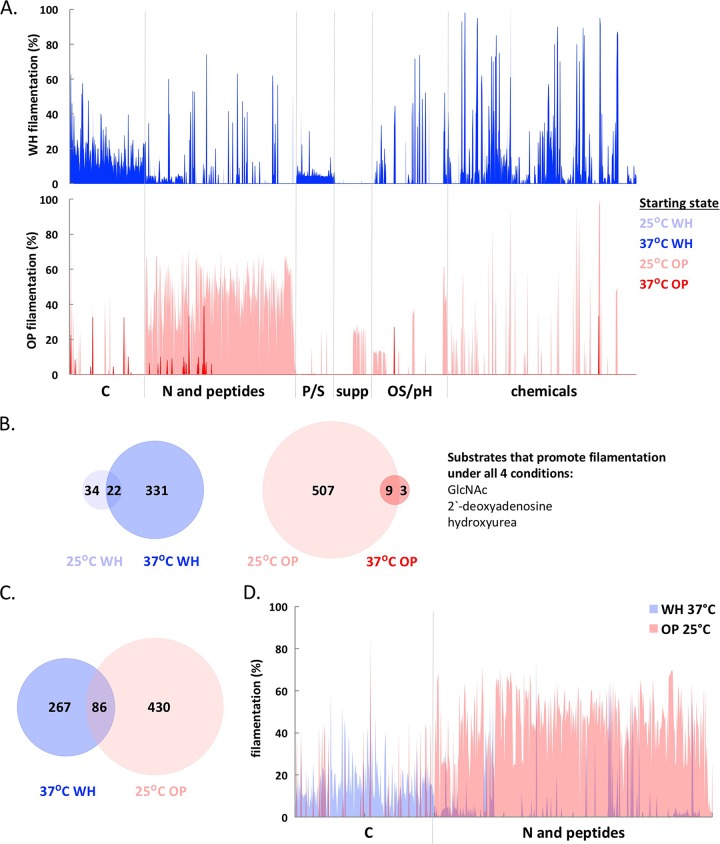
Filamentation of white and opaque cells is regulated by metabolic cues and temperature. (A) (Top) Percentage of white cells filamenting at 25°C and 37°C on different substrates. (Bottom) Percentage of opaque cells filamenting at 25°C and 37°C. (B) Overlap between conditions that induce >10% filamentation in white cells at 25°C and 37°C and between conditions that induce filamentation in opaque cells at 25°C and 37°C. Three conditions induced filamentation of both cell types at both temperatures. (C and D) Overlap between conditions that induce filamentation in white cells at 37°C and opaque cells at 25°C. Panel D highlights the divergent nutritional cues that induce filamentation at 37°C in white cells and at 25°C in opaque cells. For opaque cell filamentation data in panels A to D, only wells that contained >50% opaque cells at the end of the experiment were included.

Approximately 14% of conditions induced white cell filamentation (i.e., >10% of the population grew as filamentous cells), resulting in the formation of pseudohyphae (chains of elongated cells with constrictions between cells) or true hyphae (long, polarized cells with no constrictions between cells). Filamentation-inducing conditions included different C sources, osmotic stress substrates (OS), and “chemicals” (Fig. 4A; see Fig. S2E and S2F and Table S1D in the supplemental material). Temperature was a particularly important factor influencing filamentation—353 conditions induced white cell filamentation at 37°C compared to only 56 conditions at 25°C (Fig. S2E). Several sugars (including glucose, fructose, maltose, etc.) represented the strongest inducers of true hyphae at 37°C (Table S1D). Stimuli that induced filamentation at both temperatures included GlcNAc (and GlcNAc variants), as well as several chemicals and arginine-containing peptides (Fig. 4B; Table S1D).

### (ii) Filamentation of opaque cells.

Conditions that induced efficient opaque-to-white switching made assessing opaque cell filamentation challenging, as the morphologies of filamenting white and opaque cells are similar. For this study, “opaque filamentation” was designated when at least 50% of the cells present at the end of the experiment were opaque and growing as pseudohyphal chains or long filaments. We note that in many cases filamenting opaque cells formed pseudohyphae rather than true hyphae. Of the conditions that induced filamentation at 25°C, the majority also stabilized the opaque state, whereas conditions that induced filamentation at 37°C were associated with decreased opaque cell stability (see Fig. S2G in the supplemental material). Overall, 36% of the conditions at 25°C induced opaque cell filamentation, whereas only 0.8% of PM wells induced this morphology at 37°C (Fig. S2E and Table S1D). Opaque filamentation was most efficient in the presence of alternative N and peptide sources at 25°C, whereas GlcNAc variants, hydroxyurea, 2′-deoxyadenosine, and peptides containing glycine or alanine induced opaque filamentation at both temperatures (Fig. 4A; Fig. S2H and Table S1D).

These observations establish that distinct thermal cues favor filamentation in white and opaque cells. White cells undergo efficient filamentation at 37°C, whereas opaque cells preferentially filament at 25°C (Fig. 4; see Fig. S3C and S3D in the supplemental material). Only three substrates induced filamentation in both white and opaque cells at both temperatures—GlcNAc, hydroxyurea, and 2′-deoxyadenosine (Fig. 4B). Growth on these substrates also induced filamentation, albeit to a lesser extent, in cells from three other strain backgrounds (WO-1, P37005, and L26 [Table S1I]). This is perhaps not surprising, as strain SC5314 has been observed to filament more robustly than other clinical isolates (49). We note that GlcNAc, hydroxyurea, and 2′-deoxyadenosine are also strong inducers of the white-to-opaque switch (Fig. 3).

A comparison between the nutritional cues that induce filamentation in white cells at 37°C and opaque cells at 25°C revealed little overlap (only 24% of conditions) (Fig. 4C), illustrating that divergent cues induce the filamentation program in white and opaque cells. Some of these differences arise due to distinct responses to C and N sources; several C sources induce filamentation of white cells, whereas multiple N and peptide sources preferentially stimulate opaque cell filamentation (Fig. 4D). Stressful conditions often induced filamentation in white cells (at 37°C) and opaque cells (at 25°C), such as the presence of osmolytes or chemicals (see Table S1D in the supplemental material). Filamentous growth therefore appears to be a common response to a subset of stressful environments by both cell types. Overall, we conclude that white and opaque cells preferentially undergo filamentation at different temperatures and in response to different nutritional cues, further distinguishing the properties of the two cell types.

### Relationships between temperature, metabolism, white-opaque switching, and filamentation.

Monitoring of cell fitness, white-opaque switching, and filamentous growth across 1,440 PM conditions allowed for pairwise comparisons between these phenotypes. As noted above, white and opaque cells often display different fitness propensities (Fig. 1C), temperature dependencies (Fig. 2), switching patterns (Fig. 3), and filamentation properties (Fig. 4). We therefore used statistical correlations to define global relationships between these phenotypes. The most striking correlations are as follows.

(i) Conditions that induced switching to the opaque state at 25°C often also induced opaque cell filamentation at this temperature (see Fig. S4D and S4E in the supplemental material). This was evident for growth both on different C sources (*R*^2^ = 0.64; Pearson product-moment correlation coefficient ρ = 0.78) and on N and peptide sources (*R*^2^ = 0.73; ρ = 0.76). Furthermore, the majority of conditions that stabilized the opaque state at 25°C also induced filamentation in these cells (Fig. S4F; *R*^2^ = 0.48; ρ = 0.68). Opaque cells were therefore more likely to form filaments when growth conditions favored both their formation and their maintenance at 25°C. These results demonstrate close links between the metabolic cues affecting the white-opaque switch and those regulating the program of filamentous growth.

(ii) The fitness of white cells displayed a strong correlation with the propensity to filament on different C sources at 37°C (see Fig. S4G in the supplemental material; *R*^2^ = 0.82; ρ = 0.90). This suggests that faster growing white cells are more likely to undergo filamentation. It is possible that filamentous growth is linked to the induction of central C metabolic pathways under these conditions and cross talk with filamentation regulators such as Efg1 and Rgt1 (46, 60).

(iii) We observed an inverse correlation between opaque cell stability at 37°C and metabolic activity when grown on different osmolytes/pHs (see Fig. S4H in the supplemental material; *R*^2^ = 0.70; ρ = −0.82) or on different chemical substrates (Fig. S4I; *R*^2^ = 0.50; ρ = −0.65). This reflects, at least in part, the fact that white cells are significantly fitter than opaque cells under these growth conditions (Fig. 1C), so that switching to the white state results in faster growth of the cells.

### Hierarchical clustering establishes the central role of cell state on phenotypic properties.

Our analyses indicate that white and opaque cells often exhibit distinct phenotypes in response to external cues. To compare the phenotypic behaviors of white and opaque cells in an unbiased manner, we performed a hierarchical clustering analysis (HCA) (http://www.mathworks.com/help/stats/hierarchical-clustering.html) on the four experimental data sets (white and opaque cells grown at both 25°C and 37°C). In brief, HCA was iteratively used to compare the phenotypic output (metabolic activity/frequency of phenotypic switching/filamentation) of each possible pair of PM conditions in order to identify those that are the most similar.

**FIG 5:**
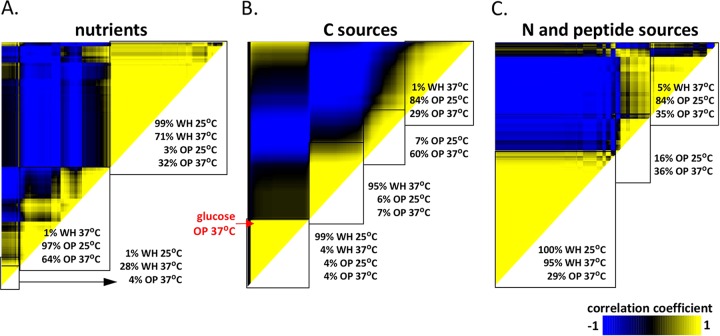
Hierarchical clustering analysis (HCA) reveals that white and opaque cells exhibit distinct phenotypic traits in response to many different nutritional cues. HCA of the four experimental data sets (white cells and opaque cells at both 25°C and 37°C) evaluates metabolic activities, filamentation phenotypes, and white-opaque switching data. HCA results are shown as heat maps populated with Pearson product moment correlation coefficients (−1 to 1) between any two conditions. The effects of different substrates on phenotypic outputs were examined for all “nutrients” (PM plates PM01 to PM08) (A), C sources (PM plates PM01 and PM02) (B), and N and peptide sources (PM plates PM03 and PM06 to PM08) (C). Division of clusters was assigned based on clustergram linkage, and only first- and second-order clusters are shown as a percentage of conditions for each starting state. Substrates with a 0 correlation coefficient generally showed no growth under these conditions.

Using this algorithm, we compared the behavior of white and opaque cells grown on different substrate categories at both 25°C and 37°C. Overall, HCA established that white cells grown under a variety of nutrient conditions clustered together, but they clustered independently of the majority of opaque cell conditions. Thus, 99% of white cells grown at 25°C and 71% of white cells grown at 37°C clustered together, but they clustered separately from 97% of opaque cells grown at 25°C and 64% of opaque cells grown at 37°C (Fig. 5A). The distinct effects of nutritional cues on white and opaque phenotypes were even more striking when examining subsets of nutrients. For C sources, HCA analysis produced four major clusters that separated along the lines of white cells grown at 25°C (99% of conditions clustered together), white cells at 37°C (95% of conditions), opaque cells at 25°C (84% of conditions), and opaque cells at 37°C (60% of conditions) (Fig. 5B). These results clearly illustrate how both cell type and temperature shape the response of *C. albicans* cells to C sources.

Growth on N and peptide sources (PM03 and PM06 to PM08) also elicited distinct responses by white and opaque cells. HCA analysis showed that 100% of white cells grown at 25°C clustered together with 95% of white cells grown at 37°C. However, separate clusters contained 100% of opaque cells grown at 25°C and 71% of opaque cells grown at 37°C (Fig. 5C). This clustering pattern reflects the dominant role of cell state in determining the phenotypes of cells grown on N sources. In particular, various N and peptide sources induce filamentation and stability in opaque cells but are poor filamentation inducers in white cells (Fig. 3A and 4A).

Similar analyses comparing the four data sets on pH and chemical/osmotic stress are detailed in Fig. S5 and Text S1 in the supplemental material. Overall, these unbiased analyses underscore how the two epigenetic states display distinct behaviors across a wide array of environmental conditions. Our studies establish that cell specialization allows distinct cell types to preferentially utilize different nutrients and to undergo filamentation in response to distinct metabolic cues.

### A complex role for glucose in regulating the white-opaque switch.

**FIG 6:**
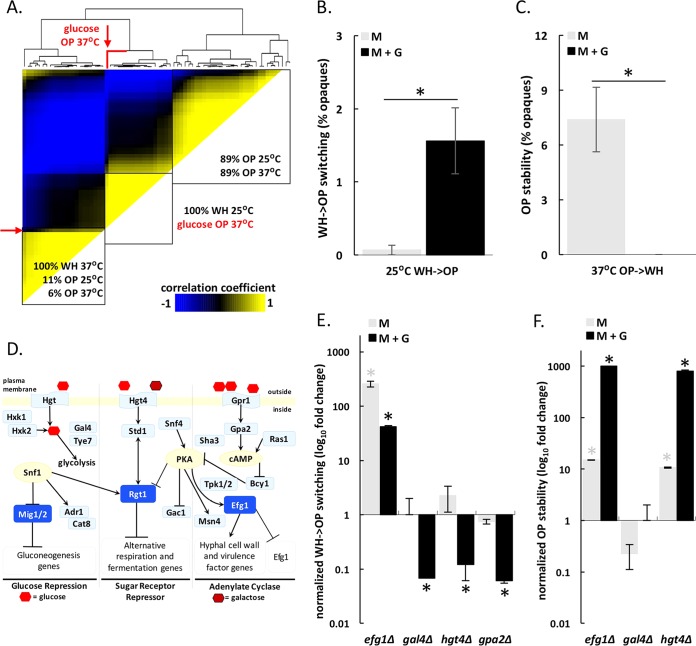
Glucose plays a complex role in regulating white-opaque transitions. (A) Hierarchical clustering analysis of the four experimental data sets (white/opaque cells at 25°C/37°C) grown on 18 different sugars including glucose, galactose, fructose, mannose, maltose, lactose, sucrose, GlcNAc variants, sorbitol, and mannitol. A clustergram is included at the top of the heat map. The location for opaque cells grown with glucose at 37°C is highlighted in red. (B and C) Impact of glucose on white-to-opaque switching at 25°C (B) and on opaque cell stability at 37°C (C). Phenotypic transitions were assessed after growth on plates containing synthetic complete medium (SC) supplemented with 1% mannitol (M) or 1% mannitol plus 1% glucose (M+G). Switching was quantified after 7 or 8 days at 22 to 25°C or 3 or 4 days at 37°C (asterisks denote significant differences [*P* < 0.05] between the M and M+G values). Results represent averaged data from four to six biological replicates. (D) Glucose sensing and signaling pathways in *C. albicans*. Pathways were adapted from reference 62 with additional components included based on homology with signaling pathways in *S. cerevisiae*. PKA, protein kinase A; cAMP, cyclic AMP. (E and F) Impact of glucose on white-to-opaque switching at 25°C (E) and opaque cell stability at 37°C (F) for *gal4*Δ, *gpa2*Δ, *hgt4*Δ, and *efg1*Δ deletion strains. Phenotypic transitions were assessed after growth on SCM or SCM+G medium for 7 or 8 days at 25°C or for 3 or 4 days at 37°C (asterisks denote significant differences [*P* < 0.05] relative to the values for control strains).

HCA analysis revealed that opaque cells grown in the presence of glucose at 37°C clustered separately from opaque cells grown on most other C sources (Fig. 5B). This was true even when comparing growth on glucose versus growth on other sugars (including fructose, mannose, sucrose, and GlcNAc variants; Fig. 6A). This was unexpected as glucose is a key regulator of metabolism and filamentation in *C. albicans* and is thought to be metabolized similarly to fructose and galactose (60–65).

To examine the effect of glucose on white-opaque switching, quantitative switching assays were performed using mannitol as a C source either in the presence or absence of glucose (G). Growth on 1% mannitol (M) did not significantly impact switching frequencies in either direction when compared to other carbohydrates (see Table S1C in the supplemental material). However, addition of 1% glucose (M+G) resulted in a ~10-fold increase in the rate of white-to-opaque switching compared to experiments performed on M alone (Fig. 6B). These effects were concentration dependent; increasing glucose levels up to 16% further increased the white-to-opaque switching frequency (Fig. S6A). The effect of glucose on switching did not correlate with the effect on growth; cells reached their maximum growth rates at glucose concentrations as low as 0.5%. Although the presence of glucose promoted white-to-opaque switching at 25°C, glucose had the opposite effect at 37°C where it destabilized the opaque state. Thus, opaque cells grown at 37°C on M alone were ~90-fold more stable than cells grown in the presence of glucose (Fig. 6C), and glucose levels as low as 0.06% destabilized the opaque state (Fig. S6C). In contrast to experiments performed at 37°C, glucose did not alter the rate of opaque-to-white switching at 25°C (Fig. S6B).

Glucose has previously been shown to modulate filamentation via signaling mechanisms in which cells respond to glucose concentrations as low as 0.01% (14, 62, 66). Given the sensitivity of opaque cells to low levels of glucose at 37°C, it is therefore likely that the destabilizing effect of glucose is mediated by high-affinity glucose signaling pathways. In contrast, glucose concentrations as high as 16% promoted the white-to-opaque switch at 25°C. This suggests that metabolic flux, or the rate at which molecules proceed through the glycolytic pathway, rather than glucose sensing *per se*, might control white-to-opaque switching frequencies under these conditions, as further discussed below.

### Genetic analysis of the role of glucose signaling pathways in white-opaque switching.

To address the mechanism by which glucose regulates the white-opaque switch, we examined components of three major glucose-sensing pathways—the glucose repression pathway, the sugar receptor repressor (SRR) pathway, and the adenylate cyclase pathway (61, 62)—for their effect on switching. We also determined whether transcription factors known to control glycolysis (67, 68) have an effect on switching. For comparison, we analyzed the phenotypes of strains lacking known transcriptional regulators of mating and the white-opaque circuit (38, 40), as well as the roles of genes with homology to *S. cerevisiae* glucose signaling components (Hxk2, Cat8, Adr1, Snf4, and Sha3).

As expected, some of the strongest effects on phenotypic switching were observed in mutants lacking components of the white-opaque circuit (e.g., *WOR1*, *WOR2*, *EFG1*, or *CZF1*), and these phenotypes were largely independent of exogenous glucose concentrations (see Fig. S6 in the supplemental material). Our analyses also implicated several glucose signaling pathways in regulating phenotypic switching. For example, deletion of components from the glucose repression pathway (*HXK2* and *GAL4*), the sugar receptor repressor pathway (*HGT4*), or the adenylate cyclase pathway (*GPA2*) reduced the frequency of white-to-opaque switching in the presence of glucose at 25°C (Fig. 6E and Fig. S6D). Hxk2 is a hexokinase acting in the first step of glycolysis, whereas Gal4 is a transcription factor involved in activating glycolysis (67, 68). These results are consistent with glycolytic flux increasing white-to-opaque switching in the presence of glucose at 25°C (Fig. S6A). However, deletion of several putative glucose signaling genes had the opposite effect on switching, so that these mutants showed increased white-to-opaque switching in the presence of glucose (Fig. S6). This result may reflect the complexity of glucose signaling pathways, as well as the fact that the precise roles of these genes have largely been inferred from other species and have yet to be established in *C. albicans*.

We also examined whether glucose signaling genes impact opaque cell stability at 37°C. Loss of *HGT4*, a glucose sensor in the sugar receptor repressor pathway, increased opaque cell stability at 37°C, both in the presence and absence of glucose (Fig. 6F). The lack of specificity for glucose at 37°C is perhaps not surprising, as Hgt4, while exhibiting high affinity for glucose, also responds to other sugars, and its effects extend over several metabolic pathways (60, 69). The same is true for *EFG1*, as loss of this gene stabilized opaque cells at 37°C (Fig. 6F). This gene is integral to the white-opaque regulatory circuit in addition to glucose sensing, indicating a pleiotropic role in *C. albicans* biology.

Finally, we examined the impact of amino acids on the white-opaque switch at 25°C. In general, the presence of specific amino acids resulted in relatively small increases or decreases in white-to-opaque or opaque-to-white switching frequencies (see Fig. S7 and Text S1 in the supplemental material). Together, these analyses indicate the complexity of the nutrient signaling pathways influencing white-opaque switching and establish prominent roles for glucose signaling pathways in determining switching frequencies.

### Opaque cells are specialized for growth at ambient temperature.

**FIG 7:**
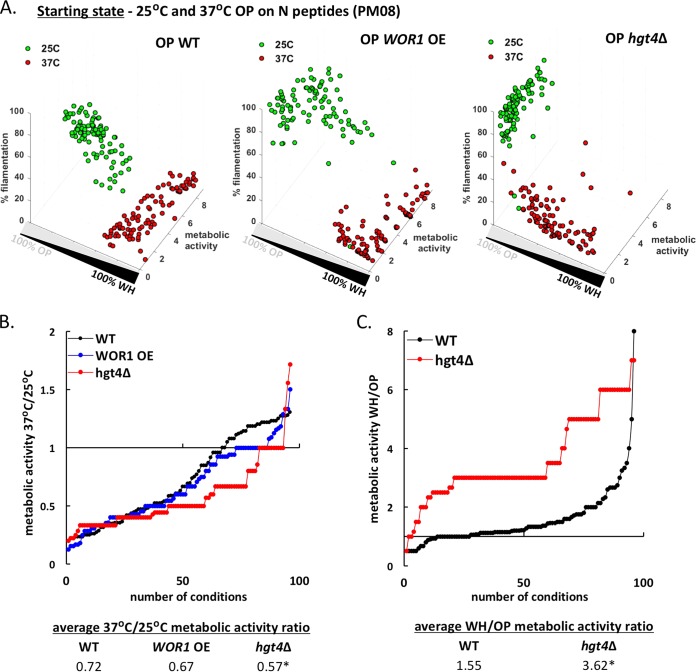
Opaque cells exhibit higher fitness, on average, at 25°C than at 37°C. (A) Phenotypic analysis of wild-type (WT), *WOR1* overexpressing (WOR1 OE), and *hgt4*Δ opaque cells grown at 25°C or 37°C for 24 h in the presence of different N peptides (PM08). (B) Ratios of metabolic activities for opaque cells at the two temperatures were calculated for each strain for each condition. The 37°C/25°C metabolic activity ratios were sorted in ascending order to illustrate differences between strains. The averages of these ratios for each strain are also listed at the bottom of the figure. Asterisks denote significant differences (*P* < 0.05) relative to the value for the WT strain. (C) Ratios of WH/OP metabolic activities of wild-type and *hgt4*Δ cells grown at 37°C on N peptide sources (PM08). The WH/OP metabolic activity ratios were sorted in ascending order, and the averages of these ratios are listed. Asterisks denote significant differences relative to the WT strain (*P* < 0.05).

Opaque cells generally exhibited lower metabolic activity at 37°C than at 25°C (Fig. 2), and we examined whether this is a consequence of cells switching to the white state or whether opaque cells are truly wired for optimal fitness at the lower temperature. To test this, we increased opaque cell stability by overexpressing *WOR1* (*WOR1* OE strain [40, 42]) or by using the *hgt4*Δ mutant that displays increased opaque cell stability (see Fig. S6E in the supplemental material). Strains were grown for 24 h on different N and peptide sources (PM08 plate) and examined for metabolic activity, opaque cell stability, and filamentation. At 25°C, wild-type (WT), *WOR1* OE, and *hgt4*Δ opaque cells were all mostly stable, although *hgt4*Δ cells displayed the highest levels of stability (Fig. 7A). At 37°C, WT and *WOR1* OE opaque cells showed frequent switching to the white state, whereas *hgt4*Δ cells showed a broader distribution of cell types, with most cells retaining the opaque state (Fig. 7A).

Next, the 37°C/25°C metabolic activity ratios were compared for WT, *WOR1* OE, and *hgt4*Δ opaque cells across all N sources. This revealed that the *WOR1* OE strain displayed a metabolic profile similar to that of the WT strain (average 37°C/25°C activity ratios of 0.72 for the WT strain and 0.67 for *WOR1* OE strain), whereas the *hgt4*Δ strain exhibited a lower 37°C/25°C ratio across multiple conditions (average 37°C/25°C activity ratio of 0.57), indicative of a bigger fitness difference between opaque cells grown at the two temperatures (Fig. 7B; see Fig. S8D in the supplemental material). We also compared the WH/OP metabolic activity ratio at 37°C for the WT and *hgt4*Δ strains (Fig. 7C). This indicated that white cells were, on average, fitter than opaque cells in both strain backgrounds but that this difference was exacerbated in the *hgt4*Δ background (WH/OP activity ratios of 1.55 for the WT strain and 3.62 for the *hgt4*Δ strain). Taken together, these data are consistent with *hgt4*Δ opaque cells exhibiting a greater fitness defect than WT cells at 37°C due to enhanced opaque cell stability at this temperature. As a control, we note that *hgt4*Δ cells did not exhibit a general fitness defect relative to WT cells when examined in the white state at 37°C (Fig. S8E).

Transcriptional analysis of white and opaque cells previously highlighted gene expression differences in carbon metabolism pathways (see Table S1E in the supplemental material) (34, 35). We therefore directly compared white/opaque cell fitness using C sources that are part of central carbon metabolic pathways (glycolysis, TCA cycle, pyruvate decarboxylation). We found that metabolic activities on these substrates were consistently lower for opaque cells than for white cells (average activity of 4.7 for white cells and 3.5 for opaque cells [Table S1F]). While high temperature did not have a significant impact on how white cells processed substrates from glycolysis or the TCA cycle, it significantly compromised the metabolism of TCA cycle substrates by opaque cells (Fig. S8F). Opaque cells grown on TCA cycle substrates had lower metabolic activities at 37°C than at 25°C (average 37°C/25°C activity ratios of 0.93 for white cells versus 0.45 for opaque cells [Table S1F]). We therefore suggest that the growth defects of opaque cells at 37°C could, at least in part, be mediated by decreased TCA cycle activity in these cell types at this temperature.

Together, the results of these experiments establish that *C. albicans* opaque cells are generally less fit than white cells at 37°C and that this difference is not due to switching of opaque cells to the white state. Furthermore, we propose that one reason for the lower fitness of opaque cells at 37°C is the decreased capacity for signaling through central metabolic pathways.

### Opaque cell stability *in vivo*.

**FIG 8:**
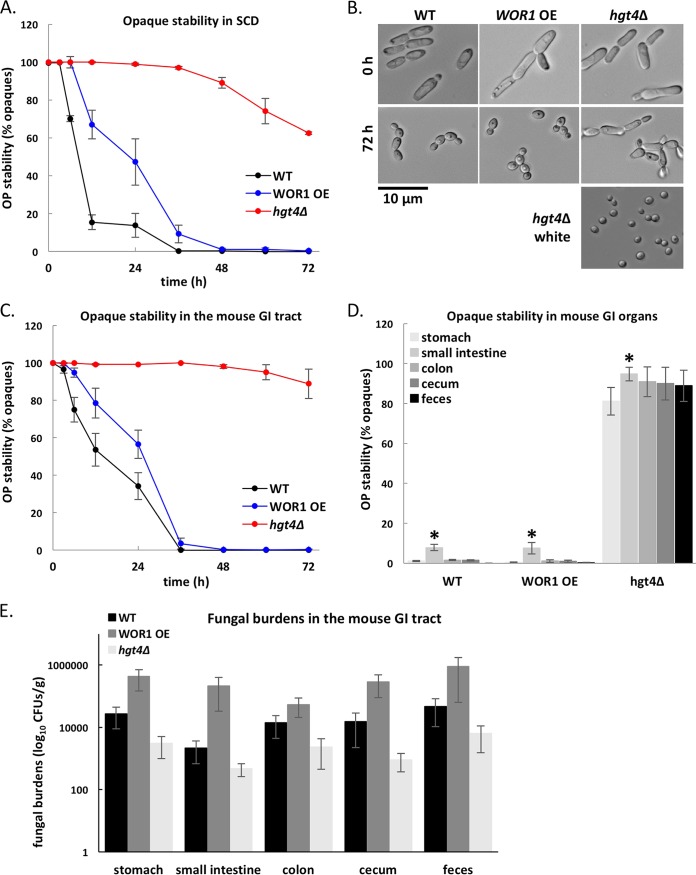
Stability of the opaque state in WT, *hgt4*Δ, and *WOR1* overexpressing cells *in vitro* and *in vivo*. (A and B) The stability of opaque cells was determined during growth in liquid SCD medium at 37°C for 3 days. Stability was assessed by plating cells onto SCD every 6 to 12 h and counting the percentage of opaque colonies on each plate (A), as well as by microscopy (B). Images of *hgt4*Δ white cells are shown for comparison. (C and D) Stability of the three opaque strains was compared in a mouse model of gastrointestinal colonization. The percentage of opaque cells in the population was monitored in mouse fecal pellets every 6 to 12 h (C) and in GI organs at 3 days (D) (asterisks denote significant differences [*P* < 0.05] between the values for the small intestine and the other GI organs). (E) Colonization levels by the three strains in the different GI organs and mouse feces were determined by calculating fungal burdens (CFU/g) after 3 days of infection (no significant differences between strains).

Opaque cells are generally unstable within the mammalian host, rapidly reverting to the white state (21, 25, 28, 70). Here, we compared the stability of opaque cells grown in standard *in vitro* conditions at 37°C with cells grown in a mouse gastrointestinal (GI) model of commensal colonization. Wild-type opaque cells were unstable under both conditions, although switching to the white state was less frequent *in vivo* than *in vitro* (Fig. 8A and C). The *WOR1* OE strain behaved similarly to the wild-type strain, undergoing frequent switching to the white state despite constitutive expression of *WOR1*. In contrast, opaque *hgt4*Δ cells were relatively stable at 37°C, with >60% of the population remaining opaque after 72 h *in vitro* and >90% of the population remaining opaque during *in vivo* colonization (Fig. 8A to C). Dissection of GI tract organs and isolation of the colonizing fungal population revealed that the relative proportions of white and opaque cells were similar to those found in fecal pellets (Fig. 8D). The small intestine consistently showed higher levels of opaque cell stability compared to other GI niches (Fig. 8D). Examination of colonization levels revealed that *hgt4*Δ fungal burdens were generally lower than those seen for the wild-type strain, whereas *WOR1* OE levels were slightly higher (Fig. 8E). The latter is consistent with the previously reported increased fitness of *WOR1* OE cells in the mouse GI tract (52). These results establish that temperature is not the only factor contributing to destabilization of the opaque state *in vivo* and that changes in nutrient signaling pathways can alter the propensity of *C. albicans* cells to adopt one phenotypic state over the other in the mammalian host.

### Metabolic differences between white and opaque cells impact competitive fitness and biofilm formation.

Thus far, the results of our experiments demonstrate that populations of white and opaque cells display preferential growth on different nutrients and at different temperatures. This suggests that either one cell type or the other will exhibit a competitive fitness advantage under select environmental conditions. To test this, we performed competition experiments starting with a 50:50 mix of white and opaque cells and analyzed the competitive fitness of the two cell states under different nutritional environments. We also examined whether differences between cell types contribute to differences in biofilm formation.

**FIG 9:**
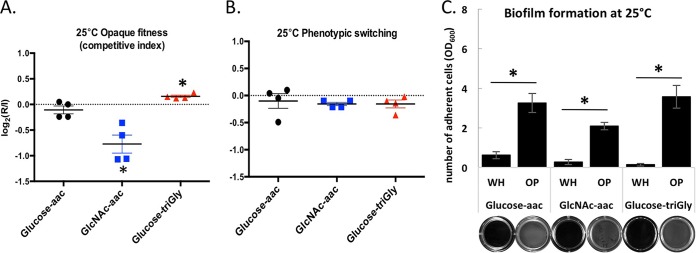
Metabolic rewiring of white and opaque cells impacts fitness and biofilm formation outcomes. (A and B) Growth in a mixed population of wild-type white and opaque cells on different C and N sources at 25°C. Cells were grown for 24 h on minimal medium with glucose and amino acids (aac), GlcNAc and amino acids, or glucose and triGly (Gly-Gly-Gly) without amino acids. Histograms show the opaque competitive index and the rate of phenotypic switching calculated as the log_2_(*R*/*I*). *R* represents the recovered opaque population after 24 h growth starting from a 1:1 white-opaque mix as the initial population (*I*). Relative changes were calculated using genetic selection (A) (either the white or opaque cell type carried a resistance marker while the other cell type did not) or visual inspection of colony morphologies (B) (by plating cells at the end of the 24-h period). Asterisks denote significant differences (*P* < 0.05) relative to the initial population. (C) Biofilm formation by white and opaque wild-type cells grown on different C and N sources at 25°C. Cells were grown on Lee’s medium with glucose and amino acids, Lee’s medium with GlcNAc and amino acids, or Lee’s medium with glucose and triGly without amino acids. Histograms show OD_600_ values following resuspension of adherent cells after 24 h of growth. The images below the bars show representative wells, and asterisks denote significant differences (*P* < 0.05) between cell types.

We examined the competitive fitness of white and opaque cells grown in media containing glucose+amino acids (aac), glucose+Gly-Gly-Gly (triGly), or GlcNAc+aac at 25°C (Fig. 9A). Growth in glucose+aac did not provide an obvious fitness advantage for cells in the white or opaque state. In contrast, growth in GlcNAc+aac resulted in white cells exhibiting a fitness advantage, whereas growth in glucose+triGly revealed a competitive advantage for cells in the opaque state (Fig. 9A). These results are consistent with the relative fitness of pure populations of white and opaque cells under these culture conditions (see Table S1C in the supplemental material). We note that growth on either GlcNAc or triGly did not result in significant levels of white-opaque switching under these conditions (Fig. 9B).

*C. albicans* biofilms are a major health concern in the clinic and are influenced both by filamentation and by the phenotypic state of the cell (71–75). While standard conditions supporting white cell biofilm formation are established (e.g., RPMI 1640 medium at 37°C), our experiments suggested alternative conditions under which opaque cells might form biofilms more efficiently than white cells. A comparison of biofilm formation at 25°C showed that opaque cells formed robust biofilms when grown in media containing glucose+aac, GlcNAc+aac, or glucose+triGly, whereas white cells did not efficiently adhere to the substrate or form biofilms under these conditions (Fig. 9C).

These experiments establish that the differential wiring of white and opaque cells gives rise to important phenotypic differences. Under most conditions, white cells exhibit increased fitness and biofilm formation over opaque cells, yet under selective conditions, opaque cells can exhibit increased fitness and biofilm formation relative to white cells.

## DISCUSSION

Epigenetic switching between white and opaque states is known to regulate niche specificity, virulence, and sexual competency in *C. albicans*. In this study, we provide an extensive analysis of the growth of white and opaque cells under various nutritional conditions at both 25°C and 37°C. As such, this work represents the first global analysis of the properties of the two cell types under diverse environmental conditions and addresses their relative fitness, their propensity to undergo filamentation, and their tendency to adopt one phenotypic state over the other. While these conditions do not mimic the natural host environment, they include growth at mammalian body temperature as well as many nutritional and stressful conditions that fungal cells encounter in the host.

Previous studies highlighted transcriptional differences between white and opaque cells, with ~17% of transcripts differentially expressed between the two cell types (34, 35). Approximately one-third of these differences were in genes assigned to metabolic pathways, suggesting that the two cell types may exhibit distinct metabolic properties (34) (see Table S1E in the supplemental material). Our study provides direct experimental support for the extensive rewiring of metabolic circuits between white and opaque cells by profiling growth under 2,880 conditions. In addition, we establish connections between the white-opaque switch, filamentous growth, and metabolic signaling pathways. The major conclusions of this study follow.

(i) White-opaque switching fundamentally alters the metabolism of *C. albicans* cells, as the two cell states are wired for optimal growth on different nutrients and at different temperatures (Fig. 1 and 2). Mathematical analysis demonstrates that the phenotypic state has a dominant effect on how *C. albicans* cells integrate environmental inputs (Fig. 5). Examination of 2,880 conditions revealed that white cells were fitter than opaque cells in 855 of these conditions, most of which were at 37°C. These results build on previous observations of differential expression of metabolic genes between white and opaque states (34, 35) and are consistent with white cells representing a “general-purpose” state capable of growth in multiple niches in the mammalian host.

(ii) There were only a limited number of conditions where opaque cells were fitter than white cells (62 out of 2,880 conditions), and these generally involved growth at 25°C rather than 37°C (Fig. 1, 2, and 9; see Fig. S8F in the supplemental material). These findings suggest that opaque cells represent a more metabolically specialized form of *C. albicans* than white cells, with a fitness advantage under select conditions. The preference for growth at lower temperatures may contribute to the enhanced ability of opaque cells to colonize host niches such as the skin, where the temperature (31.5°C) is lower than that in systemic infections (22). We also note that other host cues, including changes in O_2_ or CO_2_ levels, are also expected to impact the relative fitness of the two states, as well as switching propensities (76, 77), although these factors were not addressed in the current study.

(iii) The opaque state is relatively unstable to external perturbations, whereas the white state is generally stable. This observation supports the contention that opaque cells represent a more specialized state than white cells (see Fig. S4A in the supplemental material). Nutritional cues that induced white-to-opaque switching included established signals (e.g., hydroxyurea and GlcNAc [31, 32]), as well as novel cues such as 3-amino-1,2,4-triazole, 2′-deoxyadenosine, and multiple di- and tripeptides. Using Waddington’s analogy of epigenetic “landscapes” (78, 79), our results suggest that white cells exist in a deeper, more stable “valley” than opaque cells, making stochastic transitions to the opaque state relatively rare in most environments.

(iv) White and opaque cells undergo filamentation in response to different nutritional and thermal cues (Fig. 4). White cells displayed efficient filamentation when grown at 37°C on a variety of different C sources, whereas opaque cell filamentation was favored by different N and peptide sources at 25°C. These results extend previous observations that the circuits in white and opaque cells are differentially wired for filamentation and that distinct stimuli induce filamentation in the two states (59, 80). In addition, both cell types exhibited filamentous growth in the presence of chemical stressors, likely reflecting a shared morphological response to delayed cell cycle progression (81, 82).

(v) The regulation of the white-opaque switch shows extensive integration with that of filamentous growth. For example, nutrient cues that increased white-to-opaque switching overlapped with those that increased opaque cell filamentation. Furthermore, growth conditions that promoted the stability of the opaque state also promoted opaque cell filamentation. Thus, many of the same metabolic cues impact both phenotypic switching and filamentation. This observation is likely the direct result of white-opaque switching and filamentous growth sharing transcriptional regulators (44, 77, 83–86).

(vi) Genetic analyses identified three glucose signaling pathways that influence white-opaque switching and revealed that they can do so in the presence or absence of glucose. Glucose increased white-to-opaque switching at 25°C but decreased the stability of the opaque state at 37°C (Fig. 6). Several factors regulate this process including the sugar sensor Hgt4. Loss of Hgt4 reduced glucose-induced white-to-opaque switching at 25°C but increased opaque cell stability at 37°C (Fig. 6). These results emphasize the complex role that glucose (and other nutritional cues) play in regulating the white-opaque switch.

(vii) Modulation of metabolic pathways can influence phenotypic switching *in vivo*, as loss of Hgt4 stabilized the opaque state in a commensal model of gastrointestinal colonization (Fig. 8). This result demonstrates the importance of metabolism in determining the phenotypic state of the cell and suggests that opaque cells can be stably maintained in the host given appropriate metabolic cues.

(viii) Differential wiring of the two cell states impacts biofilm formation. White and opaque cells underwent efficient biofilm formation at different temperatures, and this process was strongly affected by nutritional cues (Fig. 9; see Fig. S9 in the supplemental material).

In summary, this study demonstrates that two epigenetic states of *C. albicans* show notable differences in fitness, filamentation, and biofilm formation. Our data support the role of white cells as a “general-purpose” phenotype, as these cell types grow well under a wide variety of nutritional conditions, particularly at 37°C. This is consistent with white cells being the default form of *C. albicans* in the mammalian host. In contrast, opaque cells represent a more metabolically specialized cell state, exhibiting greater fitness than white cells only under selective conditions. Opaque cells are the mating competent form of the species, which is thought to be relatively rare in nature (87–89), but may provide a competitive advantage under some conditions (90, 91). Our profiling data support the designation of opaque cells as being a specialized cell type, but whose properties further extend the fitness and phenotypic attributes of the organism, thereby enabling adaptation to a wider range of environmental situations. These studies also highlight the need for greater understanding of the natural niches that favor propagation of two cell states in the mammalian host.

## MATERIALS AND METHODS

### Strains and growth conditions.

The *C. albicans* strains used in this study are listed in Table S1G in the supplemental material. Unless otherwise stated, strains were grown at room temperature (22 to 25°C) in synthetic complete medium supplemented with 2% glucose (SCD) and plated on SCD agar. To analyze the role of glucose in phenotypic switching, glucose was replaced with 1% mannitol (M) or 1% mannitol plus 1% glucose (M+G) as specified.

### Strain construction.

Lists of strains, oligonucleotides, and plasmids used in this study can be found in Table S1G in the supplemental material. For the Phenotypic MicroArray (PM) panels, strains RBY717 (white) and RBY731 (opaque) were used (92). Deletion strains have either been previously reported (41, 59) or were constructed from previously reported deletion mutants in the **a**/α cell background. Deletion mutants of transcription factors were generated from an **a**/α deletion library in the SN152 background (93–95) as described in reference 86. The *hex1*Δ, *hgt6*Δ, *hgt8*Δ, and *opt4*Δ mutants were created in strain SN152 and converted to **a**/Δ strains using plasmid pJD1 (72) to delete the *MTL*α locus. Several mutants (*gac1*Δ, *gpa2*Δ, *hgt4*Δ, *sha3*Δ, *snf4*Δ, *tpk1*Δ, and *tpk2*Δ) were constructed in RZY47 (an **a**/**a** strain) (38, 41) using fusion PCR with *HIS1* and *LEU2* cassettes (95). Correct chromosomal integration of each marker and loss of the open reading frame (ORF) were verified by PCR.

### Phenotype MicroArray panels.

PM panels and reagents (inoculating fluid IFY-0 base, redox dye mix D and E) were purchased from Biolog, Inc. (Hayward, CA, USA). Other chemicals were purchased from Sigma-Aldrich (St. Louis, MO, USA) unless otherwise stated. White and opaque *C. albicans* cells were grown overnight in SCD medium at 25°C, washed twice in H_2_O, and diluted to an optical density at 600 nm (OD_600_) of 0.5. The cells were added to the inoculating fluid at a final OD_600_ of 0.01. The PM panels represent 96-well plates containing different substrates in each well including carbon sources (PM01 and PM02), nitrogen and peptide sources (PM03 and PM06 to PM08), phosphorus and sulfur sources (PM04), biosynthesis pathway end products and nutrient supplements (PM05), osmotic stress and pH substrates (PM09 and PM10), as well as different chemical substrates, toxic ions, antibiotics, and antifungals (PM21 to PM25). The various substrates can be accessed through the Biolog website (http://www.biolog.com/products-static/phenotype_microbial_cells_literature.php). In addition to each substrate, PM wells also contain the minimal components required for normal growth. The inoculating fluid for each panel was prepared according to the manufacturer’s instructions. All panels contain glucose as the main C source except for PM01 and PM02. Additive solutions and dyes were added according to Table S1G in the supplemental material. PM plates were incubated with shaking (200 rpm) at the appropriate temperature (25°C or 37°C) for either 24 h or 48 h. As the dye is reduced, a purple color is irreversibly formed which can be detected at *A*_590_ (96, 97). Reduction of this dye correlates with respiratory growth, as the production of NADH reduces the tetrazolium violet dye in a redox reaction, resulting in the formation of a purple color. Therefore, the rate of the electron flow through the respiratory chain is used as a proxy for cellular growth. To check whether kinetic curves of dye reduction corresponded to cell growth, experiments were performed on a single nutrient (glucose) in the presence and absence of dye. Wells at the end of the dynamic range were checked under the microscope to verify the accuracy of the analysis; wells with an activity index of 0 showed no growth, while those with an activity index of 9 had reached confluence. The dye reduction was measured every 2 to 4 h for analyses across the 15 PM plates and every 15 min for the genetic analyses on individual panels. Growth on the PM plates was performed in duplicate for each strain and temperature.

### PM data analysis.

The resulting dye reduction values were imported into a PM analysis software suite (DuctApe [http://combogenomics.github.io/DuctApe/]) which calculates metabolic activities (98). Five PM growth curve parameters from each data set were extracted from the raw data—length of the lag phase, slope of the curve, average height of the curve, maximum cell respiration, and area under the curve, which were used to calculate final metabolic activity parameters through k-means clustering (98). The averaged metabolic activities for wild-type white and opaque cells tested on the PM panels at both temperatures can be found in Table S1A in the supplemental material. Statistical analyses between types or subtypes of nutrients were performed using pairwise two-tailed *t* tests in Excel, and significance was assigned for *P* values of <0.05. At the end of the growth period, the PM wells were microscopically examined for switching and filamentation.

### Microscopy analyses.

Colonies were inspected, and images were collected using a Zeiss Stemi 2000-C microscope equipped with an Infinity 2 digital camera and Infinity Analyzer software (Lumenera Corporation, Ottawa, Canada). The morphology of cells in the PM panels was examined and recorded using differential interference contrast (DIC) on a Zeiss inverted microscope (Axio Observer) fitted with an AxioCam HR. Images were processed with AxioVision 4.8 (Zeiss, Germany) and Photoshop. To quantify cellular phenotypes, 200 to 400 cells were evaluated for each condition. White filamentation was defined as either white hyphae with no constrictions at the septa or white pseudohyphal cells. Opaque filamentation was defined as either opaque cells exhibiting long hyphae with no constrictions at the septa or chains of >10 opaque cells that display branching. The majority of wells showing significant phenotypes (e.g., >2% white-to-opaque switching) were also plated onto SCD medium, and colonies were examined after 5 to 7 days of growth at 22 to 25°C. All panels were also spotted onto SCD plates and phenotypes such as lack of growth, high filamentation, high switching to the opaque state, or high levels of opaque stability were determined. Three-dimensional (3D) plots of phenotypic parameters (metabolic activity, switching, and filamentation) for wild-type and mutant strains were generated using MATLAB R2015b.

### Competition assays.

Competition assays were performed by mixing together white and opaque cells in a 1:1 ratio and growing for 24 h at 25°C or 37°C. Yeast cells from overnight cultures were washed, diluted, and counted in duplicate using a hemocytometer to accurately calculate ratios. A total of 10^6^ white and opaque cells was then added to 4-ml cultures containing synthetic complete medium (SC) with 1% glucose (glucose+aac), SC with 1% GlcNAc (GlcNAc+acc), or SC without amino acids but with 1% glucose and 1% triGly (glucose+triGly). After 24 h of growth at 25°C or 37°C, cells were sonicated, diluted, and plated onto SCD and yeast extract-peptone-dextrose (YPD)-nourseothricin (NAT) plates, and grown for 3 or 4 days at 25°C before counting CFUs and scoring colony morphologies. The opaque competitive index and the rate of phenotypic switching were calculated as the log_2_(*R*/*I*), with *R* representing the recovered opaque population after 24 h growth starting from a 1:1 white-opaque mix as the initial population (*I*). The percentage of NAT^+^ resistant colonies was used to determine the ratio of white/opaque cells in starting populations, whereas the percentage of white and opaque colonies indicated the phenotypic state at the end of the 24 h competition period. Results shown represent the average of four independent experiments (two experiments with white NAT^+^ versus opaque NAT^−^ cells and two experiments with opaque NAT^+^ versus white NAT^−^ cells). Significant differences relative to starting populations are shown (*P* < 0.05).

### **Biofilm formation**.

For biofilm measurements, WT white and opaque cells were grown overnight at 25°C in SCD medium. Cells were washed and resuspended in H_2_O, and 10^7^ cells were added to 1 ml of medium per well in a 12-well plate. The medium used was Lee’s medium plus 2% glucose (glucose-aac), Lee’s medium plus 2% GlcNAc (GlcNAc+aac), and Lee’s medium without amino acids with 2% glucose and 1% triGly (glucose+triGly). The 12-well plates were incubated at 25°C or 37°C for 24 h without shaking. After 24 h, each well was gently washed twice with 1 ml of phosphate-buffered solution (PBS) to remove nonadherent cells. Biofilms were imaged using a Bio-Rad Chemi-Doc imager. Biofilm mass was determined by scraping off adherent cells, resuspending them in H_2_O, and measuring the OD_600_. Results shown represent the results of three or four independent experiments, each with technical duplicates. Significant differences between white and opaque cells are shown (*P* < 0.05).

### Hierarchical clustering.

Hierarchical clustering analysis (HCA) was performed using an algorithm that iteratively compares the output between two substrate conditions in order to find those that are the most similar. Each condition represents the unique phenotypic output defined by three parameters—metabolic activity, phenotypic switching (given by the percent opaque cells in the final population), and induction of filamentation (given by the percent filamentous cells at the end of the experiment). Phenotype similarity was assessed using a Pearson product-moment correlation coefficient (ρ), which indicates how the two conditions are related (positive or negative correlation), as well as the strength of the respective relationship (with 0 denoting the absence of a correlation and +1 or −1 denoting total correlations). The Pearson correlation between each two conditions represents the covariance of the two phenotypic outputs divided by the product of their standard deviations. The phenotypic output of any condition can be represented on a 3D plot using the coordinates given by the three phenotypes (metabolic activity, percent opaque cells, and percent filamentation), similar to the plots shown in Fig. S4A in the supplemental material. HCA groups the points on these graphs based on the distance between them.

Performing the hierarchical clustering required two independent components: (i) computing pairwise Pearson product-moment correlation coefficients (*ρ*) for the measured conditions and (ii) using agglomerative clustering to group conditions into hierarchical clusters that are similar according to their Pearson correlations.

For the first component, measurements for each of the three parameters were normalized to the range [1..100]. Then, for each pair (*i*, *j*) of conditions, the Pearson correlation coefficient *P*_*ij*_ was computed, where *P*_*ij*_ represents the covariance of the two conditions divided by the product of their standard deviations. More specifically, letting *x*_*i*_ = (*m*_*i*_, *o*_*i*_, *f*_*i*_) denote the normalized measurements of metabolic activity (*m*_*i*_), percent opaque cells (*o*_*i*_), and percent filamentation (*f*_*i*_) for the *i* condition, and similarly for the *j* condition, we can define:

$$P_{ij}=\frac{\text{Cov}[x_{i},x_{j}]}{\sigma_{i}\sigma_{j}}=\frac{\left(m_{i}-\mu_{i}\right)\text{(}m_{j}-\mu_{j})+\text{(}o_{i}-\mu_{i})\text{(}o_{j}-\mu_{j})+(f_{i}-\mu_{i})(f_{j}-\mu_{j})\;}{3\sigma_{i}\sigma_{j}}$$

where *μ_i_* and *σ_i_*, respectively denote the mean *μ_i_* = (*m_i_* + *o_i_* + *f_i_*)/3, and the standard deviation $\sigma_{i}=\sqrt{\left[{\left(m_{i}-\mu_{i}\right)}^{2}+{\left(o_{i}-\mu_{i}\right)}^{2}+{\left(f_{i}-\mu_{i}\right)}^{2}\right]/3}$ of the parameters for condition *i*. The resulting coefficient *P_ij_* always belongs to the interval [−1, 1], with *P_ij_* = 0 corresponding to absence of a correlation, whereas *P_ij_* = +1 or −1 corresponds to a total correlation.

The second component uses the coefficient *P*_*ij*_ to perform agglomerative clustering on the conditions with mean linkage clustering (unweighted pair group method with arithmetic mean [99]) as the linkage criterion. This method consists of performing the following steps: (i) initially, each condition corresponds to its own cluster; (ii) while there are at least two clusters left, pick the closest two clusters and merge them into a single one. This method therefore creates a hierarchical tree whose branches correspond to individual conditions. The algorithm is completely specified once the linkage criterion (i.e., the distance between two clusters) is defined. In our case, given two clusters $A=\{i_{1},\;i_{2},\;.\;.\;.\;,\;i_{m}\}$ and $B=\{j_{1},\;j_{2},\;.\;.\;.\;,\;j_{m}\}$, the distance between them is defined as $P_{AB}=(\overset{m}{\underset{k\;=\;1}{\sum }}\overset{n}{\underset{l\;=\;1}{\sum }}P_{i_{k}j_{l}})/mn$. An implementation of this clustering scheme available in the MATLAB Statistics and Machine Learning Toolbox was used to analyze this data set (http://www.mathworks.com/help/stats/hierarchical-clustering.html).

To verify this method, we ran the algorithm on a large uniform data set randomly generated using MATLAB and containing 250 conditions compared across all four starting states. The resulting clustergram was uniform with no delimitation into clusters, and the four starting species were equally distributed across the first- and second-order branches of the clustergram (see Fig. S5C in the supplemental material). Using this algorithm, we ran comparisons between the four starting states (white cells and opaque cells at both 25°C and 37°C) on broad ranges of substrates (nutrients and chemicals) or single types of nutrients (e.g., C sources and N sources). The outputs generated consisted of clustergrams of the main and ramifying branches (http://www.mathworks.com/help/bioinfo/ref/clustergram.html) as well as heat maps populated with Pearson correlation coefficients which allow the identification of the major groups sorted. Conditions that cluster together (i.e., have a high Pearson correlation coefficient) exhibit similar phenotypes under those conditions, inducing analogous levels of filamentation, switching, and growth. For simplicity, only the first- and second-order clusters delimited by branches of the clustergram are presented in the main figures.

### White-opaque switching assays.

White-to-opaque and opaque-to-white switching screens used a variation on a previously reported assay (31). White or opaque phase cells were inoculated into liquid SCD medium and incubated overnight at 22 to 25°C. Cultures were checked for the purity of cell types (>99%), diluted in H_2_O, and plated onto SC-based medium (SCD without glucose) at a concentration of 100 to 200 colonies per plate. For M and M+G conditions (see Fig. S6 in the supplemental material), SC agar was supplemented with 1% mannitol (M) with or without various concentrations of glucose (G). For Fig. S6, 1% glucose was added to the SCM plates (M+G). For N-related switching assays (Fig. S7), all experiments were performed on SCD agar at 22 to 25°C. For WH→OP and OP→WH switching assays at 22 to 25°C, colonies were scored for opaque sectors or full colonies after growth for 7 or 8 days. For opaque stability assays (OP→WH switching at 37°C), colonies were scored for white sectors or full colonies after growth for 3 or 4 days. Plates were scored, keeping track of the numbers of colonies of the starting cell type (*A*), colonies of the starting cell type with one or more sectors (*B*), and colonies of the other cell type (*C*). Switching frequencies were calculated as [(*B* + *C*) × 100]/(*A* + *B* + *C*), which was compared to the switching frequency of the wild-type strain. Switching assays were performed in three to six replicates, and at least 100 colonies were assessed for each replicate. For Fig. 6B and C and Fig. S6A to S6C and S7A to S7D, results shown represent averaged data from two wild-type strains in the same SC5314 genetic background (RBY717 and RBY731, the strain used in the PM experiments and the parental strain for the glucose or N deletion screen, respectively), each performed in 4 to 6 biological replicates.

### Ectopic expression assays.

Ectopic expression of a subset of regulators was achieved using the doxycycline-regulated promoter in pNIM1 or pNIM6 (29, 100). pNIM1 and pNIM6 differ only in the presence of the *TEF3* (pNIM6) or *ACT1* (pNIM1) terminators downstream of the target gene. PCR-amplified ORFs were cloned into the pNIM1 or pNIM6 vector which was then digested with ApaI and SacII and transformed into the CAY616 strain. Transformants were selected on nourseothricin (*SAT1*^+^), and correct integration was verified by colony PCR against the 5′ flank. The pNIM1 vector containing *ca*GFP (*C. albicans*-adapted GFP) was also transformed into strain CAY616, and efficient tetracycline-inducible expression was verified by monitoring GFP expression.

Switching assays for overexpressing strains were performed as follows. *C. albicans* cells were grown overnight in SCD, diluted to an OD_600_ of 0.1 in 4 ml of SCD, and supplemented with 50 µg/ml doxycycline (+dox). After 4 h of growth, 100 to 300 cells were plated on the appropriate agar plates (SCM, SCM+G, or SCD) supplemented with 50 µg/ml doxycycline. Quantification of switching events was carried out following 7 or 8 days of growth at 22 to 25°C or after 3 or 4 days at 37°C. Switching rates were compared to those of untreated controls (−dox), and between 3 and 6 independent experiments were performed for each experiment.

Statistical analysis of ectopic switching assays was performed by calculating the ratio of the +dox/−dox switching rate. Averaged ratios were defined for each strain and compared to the ratio for the wild type. In Table S1H in the supplemental material, we report the averaged ratios and standard errors of the means (SEM) for the independent experiments for each strain. For several transcription factors, two independently transformed strains were tested (Table S1H). We performed paired *t* tests (two-tailed tests) comparing (i) the switching rates of the overexpressing strains versus wild-type controls and (ii) the M and M+G conditions. Significant results (*P* < 0.05) are marked with asterisks in supplemental figures or are reported in Table S1H.

### Animal infections.

For *in vivo* commensal experiments, an antibiotic-treated murine model of commensalism was used (52). Briefly, 18 6-week-old female BALB/c mice (~18 g) from Charles River Laboratories, Inc., were housed together with free access to food and water. Mice were given standard rodent chow (FormuLab 5001; PMI Nutrition International), and their water was supplemented with antibiotics (1,500 U/ml of penicillin, 2 mg/ml of streptomycin) and 5% glucose for taste. The antibiotic treatment was initiated 4 days prior to infection, and mice remained on antibiotics for the duration of the infection. WT, *WOR1* OE, and *hgt4*Δ strains were grown overnight in SCD at 25°C, washed three times, and diluted in sterile H_2_O to a concentration of 2 × 10^8^ cells/ml. Cell morphologies were checked before infection (>99% opaque cells). Each mouse (6 per group) was orally gavaged using 20-gauge 38 mm plastic feeding tubes (Instech Laboratories, Inc.) with 10^8^ *C. albicans* cells in a 500 µl volume. Dilutions of cell suspensions were plated onto agar to confirm the inoculum. Fecal pellets were collected every 6 to 12 h and homogenized using a phosphate-buffered saline (PBS) solution supplemented with antibiotics (500 µg/ml penicillin, 500 µg/ml ampicillin, 250 µg/ml streptomycin, 250 µg/ml kanamycin, 125 µg/ml chloramphenicol, and 125 µg/ml doxycycline). After 3 days, mice were sacrificed, and gastrointestinal organs (stomach, small intestine, colon, and cecum) were harvested, weighed, and homogenized in a solution of PBS plus antibiotics. Dilutions of organ and fecal homogenates were plated on SCD agar for quantification of fungal burdens and for examination of colony morphologies. Plates were incubated at room temperature for 4 or 5 days after which colony morphologies were scored, and CFUs were counted in order to determine fungal burdens per gram of organ or fecal pellet. Data from six mice were averaged, and the groups were compared at each time point using unpaired two-tailed *t* tests.

### Statistical analyses.

Statistics were performed using Microsoft Excel 2016 (Microsoft Corporation) and MATLAB R2015b. The statistical test performed was two-tailed Student’s *t* test, and significance was assigned for *P* < 0.05. Correlation coefficients were calculated using Excel and MATLAB.

## SUPPLEMENTAL MATERIAL

Text S1  Supplemental results Download Text S1, DOCX file, 0.1 MB

Figure S1  Detailed metabolic analysis across subsets of nutrients and chemicals. (A) Metabolic activity ratios of white cells versus opaque cells (WH/OP) across substrate groups at 25°C and 37°C. (B, C, and E) Averaged WH/OP metabolic activity ratios for subtypes of C sources (B), N and peptide sources (C), and P and S sources (E). (D) Amino acid distribution among peptides preferred by white cells and opaque cells (dipeptides and tripeptides). Cell type preference was calculated based on metabolic activity ratios (white cells preferred WH/OP ratios of ≥1.5, opaque cells preferred OP/WH ratios of ≥1.5). Amino acids were classified based on how often they were present in the sequences of these peptides (shown as a percentage). (F) Metabolic activities of white (blue) and opaque (red) cells grown at different pH levels at 25°C and 37°C. (G and H) White cells and opaque cells have different sensitivities to chemicals and antifungals. (G) Averaged WH/OP metabolic activity ratios shown for different osmolytes and chemicals clustered by the type of drug (left) or mode of action (right). (H) Metabolic activities of white and opaque cells grown on different antifungal agents at 25°C and 37°C. (I and J) Impact of temperature on cell type fitness upon growth on different subsets of C and N substrates. Averaged ratios of 37°C/25°C metabolic activities of white and opaque cells across different subtypes of C sources (I) and N sources (J) (asterisks denote significant differences [*P* < 0.05] between temperatures [A, B, C, E, G] or between white and opaque cells [I, J]). Download Figure S1, JPG file, 0.4 MB

Figure S2  Impact of metabolism on white-opaque switching and filamentation. (A) Summary of the number of conditions inducing WH→OP switching (>2% opaque cells at the end of the experiment) and OP stability (>50% opaque cells at the end of the experiment) at 25°C and 37°C. The numbers of conditions with no growth over the course of the experiment are indicated in red. (B) WH→OP switching levels at 25°C and 37°C averaged across different types of substrates, shown as percent opaque cells in the wells at the end of the experiment. (C) Percentage of conditions inducing *en masse* OP→WH switching (>90% white cells at the end of the experiment) for each substrate category. (D) Opaque stability levels at 25°C and 37°C averaged across different types of substrates and shown as percent opaque cells in the wells at the end of the experiment. Asterisks denote significant differences (*P* < 0.05) between the values for cells grown at different temperatures. (E) Summary of the number of conditions inducing white filamentation (>10% of cells at the end of the experiment) and opaque filamentation (>50% opaque cells present and >10% filamentation) at 25°C and 37°C. The numbers of conditions with no growth over the course of the experiment are indicated in red. (F) Filamentation levels at 25°C and 37°C in starting white populations averaged across different types of substrates. (G) Among conditions with >10% filamentation in “opaque starting wells,” those filamenting at 25°C or 37°C were associated with high or low opaque cell stability, respectively. (H) Opaque cell filamentation levels at 25°C and 37°C in “starting opaque populations” averaged across different types of substrates. Only wells that displayed >50% opaque cells at the end of the experiment were included in these statistics. For panels B and D, asterisks denote significant differences (*P* < 0.05) between the values for cells grown at different temperatures. Download Figure S2, JPG file, 0.3 MB

Figure S3  Representative microscopy images of wild-type *C. albicans* cells displaying white-to-opaque switching at 25°C (A), opaque stability at 37°C (B), white filamentation at 37°C (C), and opaque filamentation at 25°C (D). Download Figure S3, JPG file, 0.4 MB

Figure S4  Phenotypic diversity as a function of cell state, filamentation, and temperature. (A) White (left) and opaque (right) wild-type cells grown under 1,440 conditions at 25°C and 37°C plotted in a 3D space defined by fitness (metabolic activity), cell state (percent opaque cells), and degree of filamentation (percent filamentation). (B) No simple correlation defines the relationships between any two parameters of the three phenotypes examined (metabolic activity, white-opaque switching, and filamentation) across all substrates tested. (C) Overlap between conditions that induce switching (>2% opaque cells) and filamentation (>10%) in white cell starting populations or between conditions that stabilize the opaque state (>50% opaque cells) and induce filamentation (>50% opaque cells and >10% filamentation) in opaque starting populations. (D to I) Relationships between different phenotypic parameters (metabolic activity, white-opaque switching, and filamentation). Correlations between WH→OP switching and OP filamentation on C sources at 25°C (D) (PM01 and PM02) and on N and peptide sources at 25°C (E) (PM03 and PM06 to PM08). Note that for these panels, the percent opaque scale goes only to 40%. (F) Correlation between OP stability and filamentation on N and peptide sources at 25°C (PM03 and PM06 to PM08). (G) Correlation between metabolic activity and filamentation of white cells on C source plates at 37°C (PM01 and PM02). Correlations between metabolic activity and opaque stability at 25°C on osmolytes and pH substrates (H) (PM09 and PM10) and chemical substrates (I) (PM21 to PM25). All trend lines were fitted to polynomial regressions. Download Figure S4, JPG file, 0.6 MB

Figure S5  Hierarchical clustering of the OS/pH (PM09 and PM10) and chemical substrates (PM21 to PM25) conditions for the four starting states. Clustering was based on calculating Pearson product moment coefficients (ranging from −1 to 1) between any two conditions. Division of clusters was assigned based on resulting clustergram linkage, and only first- and second-order clusters are shown as a percentage of each starting state. Blocks of black areas represent substrates with a 0 correlation coefficient, and for most cases, these represent wells with no growth. (C) Hierarchical clustering on a uniform set of random parameters for metabolic activity, switching (percent opaque cells), and filamentation (percent) for 250 conditions of each of the four starting states (total of 1,000 conditions). The random phenotypic data set was generated using MATLAB and was used as a control for the clustering analysis. Clustering was based on calculating Pearson product moment coefficients (ranging from −1 to 1) between any two conditions. Division of clusters was assigned based on resulting clustergram linkage, and only first- and second-order clusters are shown as a percentage of each starting state. Clustergram and heat maps for this random data set show the absence of clustering and that the four starting states group uniformly with each other. Download Figure S5, JPG file, 0.3 MB

Figure S6  Impact of glucose on phenotypic transitions. Wild-type cells were grown in the presence of increasing glucose concentrations (0 to 16%), 1% mannitol (M), or 1% glucose plus 1% mannitol (M+G), and phenotypic switching rates were assayed on SC plates. Results represent averaged data performed in four to six biological replicates. (A to C) WH→OP switching was monitored at 25°C (A), whereas OP→WH switching was monitored at both 25°C (B) and 37°C (C). Results are shown as percent opaque cells (A) or percent white cells (B and C), and asterisks denote significant differences (*P* < 0.05) relative to the value for cells grown in 2% glucose condition (marked in red). (D to G) Genetic screens to determine the impact of glucose-related components on white-opaque switching. The impact of gene deletion or overexpression of components of the glucose sensing pathways, the white-opaque transcriptional circuit, or the mating pathway on phenotypic transitions was monitored on SCM and SCM+G media. (D and E) Changes in white-opaque switching (at 25°C) and opaque cell stability (at 37°C) for deletion strains are shown as percent opaque cells. (F and G) Changes in white-to-opaque switching (at 25°C) and opaque cell stability (at 37°C) for doxycycline-inducible overexpressing strains are shown as log_10_ fold induced changes relative to the values for no-treatment controls (+dox/−dox). Asterisks denote significant differences (*P* < 0.05) relative to the value for the parental control strain containing an empty vector (D, E, F, G). Download Figure S6, JPG file, 0.3 MB

Figure S7  Impact of modulating the amino acid source on white-opaque phenotypic switching. (A and B) Phenotypic switching at 25°C of wild-type *C. albicans* cells grown either with or without a 2% amino acid mixture. Results represent averaged data from four to six biological replicates. Histograms show percent opaque cells (A) or percent white cells (B) following growth on SCD plates for 7 or 8 days at 25°C (asterisks denote significant differences [*P* < 0.05] relative to the cells grown without amino acids). (C and D) WH→OP (C) and OP→WH (D) phenotypic switching of wild-type cells grown on different amino acid sources. Histograms show phenotypic switching rates following growth on SCD plates for 7 or 8 days at 25°C (asterisks denote significant differences [*P* < 0.05] relative to cells grown in the 2% amino acid condition). (E to H) Impact of modulating N metabolism components on phenotypic transitions. Impact of disrupting N metabolism components on white-to-opaque (E) and opaque-to-white switching (F) at 25°C. Histograms show switching rates normalized to the rate of the wild-type control strain (on a log_10_ axis) and based on percent opaque (E) or white (F) colonies following growth on SCD plates for 7 or 8 days at 25°C. (G and H) Impact of overexpressing N metabolism components on white-to-opaque switching (G) and opaque-to-white switching (H) at 25°C. Histograms show log_10_ fold induction (+dox/−dox) in phenotypic switching rates following growth on SCD plates for 7 or 8 days at 25°C (asterisks denote significant differences [*P* < 0.05] compared to the value for the parental control strain [E, F, G, H]). Download Figure S7, JPG file, 0.2 MB

Figure S8  (A to C) Contributions of *EFG1*, *HGT4*, and *STP2* to the coordinated regulation of metabolism, filamentation, and phenotypic switching in *C. albicans*. For each analysis, the top panel shows the metabolic activity of strains, and the bottom panel shows the phenotypes of cells compared for metabolic activity, phenotypic switching, and filamentation. (A) Comparison of wild-type and *efg1Δ* white cells grown on different C substrates (PM01 and PM02) at 37°C. (B) Analysis of wild-type and *hgt4*Δ opaque cells grown on different C substrates (PM01 and PM02) at 37°C. (C) Analysis of wild-type and *stp2*Δ white cells grown on different N substrates (PM08) at 25°C. (D to F) Impact of temperature on opaque cell growth at 37°C. (D) Analysis of temperature effects on opaque cell fitness in strains with different opaque cell stability levels. Heat map of metabolic activity of WT, *WOR1* OE, and *hgt4*Δ opaque cells grown at 25°C and 37°C for 24 h on N peptide plates (PM08). (E) Heat map of metabolic activity of WT and *hgt4*Δ white and opaque cells grown at 37°C for 24 h on N peptide plates (PM08). Metabolic activity is represented on a scale from blue (no growth [metabolic activity value of 0]) to yellow (maximum growth [metabolic activity value of 9]). (F) Schematic representation of the *C. albicans* TCA cycle, showing the 37°C/25°C metabolic activity ratios on substrates that are part of the central C metabolic pathway. Ratios corresponding to white cells (blue) are significantly higher than those of opaque cells (red), reflecting the decreased functioning of this pathway in opaque cells at 37°C (*P* < 0.05). Download Figure S8, JPG file, 0.5 MB

Figure S9  Metabolic rewiring of white and opaque cells impacts fitness and biofilm formation outcomes. (A and B) Growth in a mixed population of wild-type white and opaque cells on different C and N sources at 37°C. Cells were grown on minimal medium with glucose and amino acids, GlcNAc and amino acids, or glucose and triGly without amino acids. Histograms show the opaque cell competitive index and the rate of phenotypic switching calculated as the log_2_(*R*/*I*). *R* represents the recovered opaque population after 24 h of growth starting from a 1:1 white-opaque mix as the initial population (*I*). Relative changes were calculated using genetic selection (A) (either white or opaque cells carried a resistance marker) or visual inspection of colony morphologies (B) (by plating cells at the end of the 24-h period). Asterisks denote significant differences (*P* < 0.05) relative to the values for the initial population. (C) Biofilm formation by white and opaque wild-type cells grown on different C and N sources at 37°C. Cells were grown on Lee’s medium with glucose and amino acids, Lee’s medium with GlcNAc and amino acids, or Lee’s medium with glucose and triGly without amino acids. Histograms show OD_600_ values following resuspension of adherent cells after 24 h of growth. Images below show representative wells, and asterisks denote significant differences (*P* < 0.05) between the values for cell types. (D) Differential integration of metabolic and thermal cues by *C. albicans* white and opaque cells. Schematic summarizing several of the main findings of this study. Formation of both cell types (white/opaque) as well as their specific filamentation programs are favored by distinct cues—they are induced by different metabolic conditions and are wired for growth at different temperatures. Besides known components of the white-opaque circuit, we also highlight novel regulators impacting the switch. Certain substrates have complex roles in modulating phenotypic transitions in more than one direction (e.g., glucose, amino acids, and GlcNAc). Download Figure S9, JPG file, 0.3 MB

Table S1  (A) Growth of white and opaque cells at 25°C for 48 h and 37°C for 24 h on diverse nutrients and chemical substrates. Metabolic activity indexes were obtained using the DuctApe suite (http://combogenomics.github.io/DuctApe/) and represent the average values of two independent experiments. Averaged metabolic activities for each type of plate as well as significant differences between white cells and opaque cells are highlighted for both temperatures. Across substrates, opaque cells were more fit than white cells (WH/OP ratio of <0.5) on 52 conditions at 25°C and 10 conditions at 37°C. Similarly, 33 conditions at 25°C and 56 conditions at 37°C promoted increased fitness of white cells relative to opaque cells (WH/OP ratio of ≥5). Separate analyses comparing white and opaque metabolic activities at 25°C for 48 h and 37°C for 24 h on diverse chemical substrates and antifungals are also included. (B) Impact of temperature on C and N/peptide metabolism. Certain C and N sources are metabolized more efficiently at either higher or lower temperatures irrespective of cell type. Data represent ratios of 37°C/25°C metabolic activities for white and opaque cells on C source plates (PM01 and PM02) or N and peptide source plates (PM03 and PM06 to PM08) after growth for 24 h. (C) Three phenotypes, metabolic activity, filamentation (percent), and phenotypic switching (percent opaque cells), are compared across all 15 PM plates and for all four starting states (white cells at 25°C, white cells at 37°C, opaque cells at 25°C, and opaque cells at 37°C). Filamentation and phenotypic switching were assayed for each PM well by microscopy, and data represent the averages of two independent experiments. (D) Analyses showing conditions that induce >2% white-to-opaque switching, conditions that stabilize the opaque state (>50% opaque cells at the end of the experiment), conditions that induce >10% filamentation in white cells, and conditions that induce >10% filamentation in opaque starting populations at both temperatures. Conditions that stabilize the opaque cells (>50% opaque cells at the end of the experiment) and induce >10% filamentation are highlighted separately. Analyses also include the overlap between conditions that induce filamentation (>10%) in white cells at 37°C and those that induce filamentation (>10%) in opaque cells at 25°C. (E) Summary of genes regulated by cell type according to transcriptome sequencing (RNA-Seq) data (35). Genes significantly up- or downregulated are shown along with the main important GO processes and functions annotated for them (CGD). Note that metabolic genes and those involved in transport are differentially regulated between the white and opaque forms. (F) Impact of cell type and temperature on the functioning of central C metabolic pathways. Metabolic activities on substrates belonging to these pathways are compared between cell types and between temperatures, with significant differences highlighted for *P* values of <0.05. (G) Strains, oligonucleotides, plasmids, and concentrations of PM plate additives used in this study. (H) Phenotypic switching data on strains overexpressing C and N metabolism regulators. Changes in white-opaque switching (at 25°C) and opaque stability (at 37°C) are represented as fold induction changes relative to no-doxycycline treatment controls (+dox/−dox). Independently transformed strains containing the same transcription factor plasmid are indicated by “-a” and “-b”. For C metabolism regulators, *t* tests show comparisons between the M and M+G conditions with *P* < 0.05 being considered significantly different. For N metabolism regulators, *t* tests show comparisons against the wild-type (WT) strain with *P* < 0.05 being considered significantly different. (I) Phenotypic analysis of white and opaque cells from four different strain backgrounds (SC5314, WO-1, P37005, and L26). Metabolic activity, the percentage of filamentous cells, and phenotypic switching (percent opaque cells) were examined. Data represent the averages of two independent experiments. Table S1, XLSX file, 0.6 MB
