# Supplementary material for: Phenotypic Profiling Reveals that Candida albicans Opaque Cells Represent a Metabolically Specialized Cell State Compared to Default White Cells
Source: mBio. 2016 Nov 22;7(6):e01269-16. doi: 10.1128/mBio.01269-16 (PMC5120136; doi:10.1128/mBio.01269-16)
Supplement: Text S1 — Supplemental results Download [file mbo006163081s1.docx]

**Supplemental results**

**The metabolic profiles of white and opaque cells - analysis of different types of nutrients**

We performed a detailed analysis of the metabolic properties of white and opaque cells for each PM category. For all the different nutrient categories, white cells generally displayed higher metabolic activities than opaque cells at both 25°C and 37°C (average WH/OP metabolic activity ratio ≥1, Fig. S1A). This difference was particularly striking for growth on different C sources and N peptides at 37°C. For example, the average WH/OP ratio was >2.5 for cells grown on a variety of carbohydrate, carboxylic acid or amino acid substrates (Fig. S1B). White cells also showed higher fitness than opaque cells when grown on different N sources (e.g., amino acids, dipeptides, and tripeptides), and this difference was greater at 37°C than at 25°C (Fig. S1C). Interestingly, the WH/OP metabolic activity ratio was ~1 for dipeptides and tripeptides at 25°C (Fig. S1C), which prompted a closer look at these substrates. This analysis revealed that amino acids such as arginine were exclusively present in the peptides favored by white cells, while nonpolar aliphatic amino acids such as alanine, glycine, valine or leucine were preferentially distributed among peptides favored by opaque cells (Fig. S1D).

White cells also showed greater fitness than opaque cells when grown on P and S substrates, although this difference was more apparent at 25°C than at 37°C (Fig. S1E). Analysis of growth at different pHs revealed that white cells grew better than opaque cells across the entire pH range at both 25°C and 37°C, with the highest fitness (metabolic activity ≥7) of white cells at 37°C extending between pH 3.5 and 7, and the highest fitness of opaque cells at 37°C between pH 4.5 and 6 (Fig. S1F). This indicates opaque cells are not as flexible as white cells with respect to pH values, and potentially limits the niches where opaque cells could optimally grow in in the host.

We also examined differences in growth in the presence of various osmolytes or ‘chemical stressors’ and found that white cells consistently grew better than opaque cells under stress conditions (average WH/OP ratio ≥1, Fig. S1G). Some of the largest differences between white and opaque cells at 25°C were insensitivity to chemicals that target metabolism (e.g. 2-deoxy-D-glucose, caffeine) and amino acid/protein synthesis (e.g. doxycycline, cycloheximide, Blasticidin S). A closer look at the antifungal agents present in the PM plates reinforced these trends as opaque cells were significantly more susceptible to all agents except cycloheximide (Fig. S1H). In contrast, white cells were able to grow in the presence of several drugs, including cycloheximide, 5-fluorocytosine, and azoles such as propiconazole, 3-amino-1,2,3-triazole (3-AT) (Fig. S1H).

**Global relationships between temperature, metabolism, white-opaque switching and filamentation**

White and opaque cells exhibited striking phenotypic differences when compared across conditions including different fitness propensities (Fig. 1C), temperature dependencies (Fig. 2), switching patterns (Fig. 3) and filamentation properties (Fig. 4). Three phenotypic attributes (fitness, filamentation and switching) were plotted onto 3D graphs to provide a global comparison of these traits between the two cell types (Fig. S4A), as well as pairwise comparisons performed between these phenotypes (Fig. S4B). These graphs revealed:

(1) The dominant role that temperature plays in regulating the white-opaque switch and cellular phenotypes under diverse experimental conditions. At 37°C, opaque cells switched *en masse* to the white state under a wide variety of conditions, whereas at 25°C many conditions (56%) supported stable propagation of the opaque state (Fig. S4A). We also found that white-to-opaque switching was low under most conditions, with only a small subset (8%) displaying switching to the opaque state, and these conditions were predominantly at 25ᴼC (Fig. S4A). Furthermore, filamentation of white cells was often associated with growth at 37°C, whereas opaque filamentation was associated with growth at 25°C (Fig. S4A).

(2) A significant subset of conditions (22 out of 56) that induced filamentation in white cells at 25°C also induced switching to the opaque state, and these wells often displayed opaque cell filamentation as well (Fig. S4C). In addition, the majority of conditions that stabilized cells in the opaque state at 25°C also induced opaque cell filamentation (516 out of 813, Fig. S4C). Together, these results further establish close links between the regulation of the white-opaque switch and the regulation of opaque filamentous growth.

(3) Additional analyses revealed correlations between white-to-opaque switching and filamentation, opaque cell stability and filamentation, growth rates and filamentation, as well as between growth rates and opaque-to-white switching. These were most apparent for growth on subsets of nutrients and are detailed in Fig. S4D-I and in the main text.

Taken together, these observations illustrate that complex relationships exist between temperature, metabolism, the white-opaque switch and filamentous growth. In particular, they strengthen previous observations that the programs regulating the white-opaque switch and filamentation are intertwined. They also further establish the distinct properties of the two phenotypic states in response to their environment.

**Hierarchical clustering analyses of cells grown on OS, pH and chemical substrates**

We used hierarchical clustering analysis (HCA) to further compare the phenotypic outputs of the four experimental datasets (WH 25°C, WH 37°C, OP 25°C and OP 37°C) during growth on different PM conditions. As discussed in the main text, HCA consistently demonstrated that opaque cells clustered away from white cells when grown in a variety of nutrients. Similarly, growth on OS and pH substrates resulted in 72% and 41% of opaque cells (at 25°C and 37°C, respectively) clustering away from 66% and 74% of white cells (at 25°C and 37°C, respectively) (Fig. S5A). The same trends were seen when cells were grown on PM ‘chemicals’. Here, 86% of opaque cells at 25°C and 53% of opaque cells at 37°C clustered away from 77% of white cells at 25°C and 50% of white cells at 37°C (Fig. S5B). Thus, the biggest differing in clustering was observed between white cells at 37°C and opaque cells at 25°C, consistent with these cell states exhibiting very distinct phenotypic properties at the two temperatures. Overall, HCA reveals that cell state and temperature dominate how *C. albicans* cells respond to chemical cues in their environment.

**Genetic analysis of the role of glucose in regulating white-to-opaque switching**

We used a collection of deletion (Fig. S6D-E) and ectopic expression (Fig. S6F-G) strains to examine the role of glucose on the white-opaque switch. These included components of the glucose response network as well as regulators of the white-opaque switch and mating. In *C. albicans*, glucose is sensed by at least 3 pathways (glucose repression, sugar receptor repression and adenylate cyclase pathways) (Fig. 6D). Each pathway utilizes a unique signal transduction cascade, but extensive cross-talk also occurs between these pathways (extensively reviewed in (61, 62)).

In the glucose repression pathway, sugars are sensed via Hgt transporters and glycolysis is mediated via the action of the hexokinase Hxk2. Glycolysis then inactivates the Snf1 kinase, which enables Mig1/Mig2 to repress genes involved in gluconeogenesis and the adenylate cyclase pathway. In the sugar receptor repression pathway, Hgt4 senses extracellular glucose or galactose, and the signal is transduced via the degradation of Std1 and phosphorylation of Rgt1 by PKA, rendering Rgt1 inactive. This leads to the derepression of alternative respiration and fermentation genes. The adenylate cyclase pathway functions through the activation of Gpa2 or Ras1 which activate Cyr1 and lead to inactivation of the Bcy1 inhibitory subunit of PKA. Activated PKA then phosphorylates Efg1, which activates hypha-specific genes, induces glycolytic genes and represses genes essential for oxidative metabolism ((46, 61, 62)). The *HXK1*, *CAT8*, *ADR1*, *SNF4* and *SHA3* genes included in our analyses may also operate in these pathways based on studies in *S. cerevisiae*, but their roles have not been established in *C. albicans*.

The different strains were tested for white-to-opaque switching at 25°C and for opaque stability at 37°C on media containing 1% mannitol (M) or 1% mannitol + 1% glucose (M+G). In wild type cells, the presence of glucose had seemingly paradoxical effects on switching; glucose increased the frequency of white-to-opaque switching at 25°C, whereas it rapidly destabilized the opaque state at 37°C. We found that deletion of a key component (*HXK2*, *HGT4*, or *GPA2*) from any of the three glucose-signaling pathways abolished the stimulatory effect of glucose on white-to-opaque switching (Fig. S6D). Deletion of *GAL4* and *MIG1* genes had a similar effect on switching, as *gal4Δ* and *mig1Δ* cells were significantly reduced in their ability to switch to opaque in the presence of glucose (Fig. S6D). In the absence of glucose, these mutants displayed white-to-opaque switching levels similar to those of the wild type strain. The above regulators act to repress gluconeogenesis in response to exogenous glucose (68, 101) and we postulate that all three glucose-sensing pathways act as mediators of glucose-induced white-to-opaque switching. This is in agreement with observations that white-to-opaque switching is highly sensitive to glucose concentrations (Fig. S6A), suggesting that flux through the glycolytic pathway regulates this process.

In contrast to experiments with *HXK2*, *HGT4*, or *GPA2*, deletion of several downstream components of the glucose-sensing pathways had the opposite effect on switching, resulting in increased levels of glucose-dependent white-to-opaque switching (Fig. S6D). These genes included *HEX1* (glucose repression pathway), *GAC1* (sugar receptor pathway), and *RAS1* and *SHA3* (adenylate cyclase pathway). These results may reflect both the complexity of these signaling pathways, which exhibit extensive crosstalk, as well the fact that the precise roles of these genes have not been established in *C. albicans* glucose signaling.

We also tested doxycycline-inducible strains that overexpress transcription factors acting in the signaling pathways described above. As expected, overexpression of known white-opaque and mating regulators *WOR1*, *WOR2*, *CZF1* or *CPH1* increased white-to-opaque switching frequencies both in the presence and absence of glucose (Fig. S6F). Similarly, overexpression of *EFG1* decreased switching to the opaque state, consistent with its established role in promoting the white state (Fig. S6F). Interestingly, overexpression of *RGT1* also reduced white-to-opaque switching in the presence of glucose (Fig. S6F). Rgt1 is a transcription factor that acts downstream of Hgt4 to repress expression of alternative respiration, fermentation and *HGT* genes (60, 69); the present data suggests that increased expression of Rgt1 limits the effect of glucose on switching, possibly via its transcriptional repressing activity (60, 69). We also note that overexpression of *GAL4* (part of the glucose repression pathway) increased white-to-opaque switching, both in the presence and absence of glucose (Fig. S6F). This is consistent with the fact that deletion of *GAL4* repressed the switch (Fig. S6D).

Taken together, genetic analyses implicate components of all 3 glucose-signaling pathways in promoting glucose-dependent white-to-opaque switching at 25°C. This includes: (1) glycolytic flux and signaling through Hxk2-Gal4 as part of the glucose repression pathway, (2) roles for Hgt4 and Rgt1 in glucose sensing as part of the sugar repression receptor pathway, and (3) requirement for Gpa2 to promote switching in the presence of glucose via the adenylate cyclase pathway. The Efg1 transcription factor is also implicated in the latter pathway, although this pleiotropic transcription factor is also integral to the white-opaque transcriptional circuit.

**Genetic analysis of the role of glucose in regulating opaque cell stability at 37°C**

Glucose plays a complex role in regulation of the white-opaque dichotomy. As discussed above, it promotes white-to-opaque switching at 25°C, but at 37°C addition of glucose destabilized the opaque state resulting in opaque-to-white switching (Fig. 6B-C). In wild type cells, 90% of opaque cells switched to the white state in the absence of glucose (M). This instability was increased in the presence of glucose (M+G medium), with 100% of opaque cells switching to white by the end of the 3-day experiment (Fig. 6B). Deletion of several glucose-signaling components (*MIG1*, *MSN4* and *RAS1*) significantly increased the stability of opaque cells at 37°C in the absence of glucose, indicating that they can affect switching in a glucose-independent manner (Fig. S6E). Deletion of the glucose sensor *HGT4*, or the white-opaque circuit/mating genes *EFG1*, *CZF1*, *CPH1*, or *CPH2,* had more striking effects on stabilization of the opaque form at 37°C, and this stabilization was again independent of whether glucose was present or not (Fig. S6E).

Overexpression of several target genes also influenced stability of the opaque state at 37°C. The transcriptional regulators of the white-opaque circuit had major effects on stability; *EFG1* overexpression further destabilized the opaque state at 37°C, whereas increased expression of *WOR1* and *WOR2* resulted in the biggest increase in opaque cell stability (Fig. S6G). Overexpression of the glucose-signaling genes *GAL4*, *CAT8* and *STD1* also increased opaque cell stability (both in M and M+G conditions), consistent with the phenotypes of their corresponding deletion strains. In contrast, overexpression of *MIG1* and *MSN4* had opposite effects to those predicted by the phenotypes of their corresponding deletion strains, again highlighting the complexity of the sugar response network.

These observations establish that white-to-opaque switching at 25°C and opaque stability at 37°C are influenced by both glucose-signaling pathways and glucose metabolism, and that these signaling pathways can affect the switch in a glucose-independent manner. We further note that ectopic gene expression did not always produce the opposite phenotypic effect to the corresponding gene deletion mutant, in line with previous genetic screens on *C. albicans* transcription factors regulating the white-opaque switch (86). This illustrates the value of screening both deletion and ectopic expression mutants, as well as the complexity of white-opaque switching regulation.

**The role of nitrogen and amino acid cues in phenotypic switching**

We more closely examined the effect of amino acids on phenotypic switching to determine how nitrogen (N) and related nutrients impact the white-opaque switch. Based on results from the PM assays, we compared the standard 2% amino acid mixture (16 amino acids that constitutes the base N source for SCD medium), with amino acids that gave rise to significant effects in PM assays, such as fitness differences or induction of phenotypic switching or filamentation (see Fig. 2-4).

We found that the base amino acid mix stimulated both white-to-opaque switching and opaque-to-white switching by at least 3-fold relative to medium lacking any amino acids (Fig. S7A-B). This indicates that amino acids have complex roles in regulating the white-opaque switch. Next, we examined phenotypic switching rates for cells supplemented with various single amino acids or amino acid mixtures (e.g., a mix of 2% glycine + 2% proline). Supplementation with only arginine, ornithine or proline decreased white-to-opaque switching relative to the standard 2% amino acid mix, while supplementation with 2-4% glycine increased the frequency of this transition (Fig. S7C). In contrast, none of these amino acids promoted the opposite opaque-to-white switch, with switching rates similar to the no amino acid control (Fig. S7D). These results indicate that the presence of specific amino acids modulates switching frequencies and could therefore impact the phenotypes present in a population.

We next screened candidate genes to test potential pathways mediating the role of N and amino acid metabolism on switching. We examined the oligopeptide transporter Opt4, the amino acid regulated transcription factor Stp2, two transcription factors involved in N degradation and N starvation (Dal81 and Gln3, respectively), the master regulator of the amino acid response Gcn4, as well as Rpn4 (controlling proteasome genes) and Stp4 (involved in filamentation and possibly uptake of amino acids) (18, 102-108) (Fig. S7). Strains overexpressing *EFG1* and *WOR1* transcription factors were included as controls in these assays, which were performed on SCD at 25°C. The *rpn4*∆ and *stp2*∆ mutants showed increased switching to opaque (10-20 fold, Fig. S7E) while *rpn4*∆ also showed decreased switching to white (Fig. S7F). Consistent with these findings, overexpression of both of these genes increased opaque-to-white switching (Fig. S7H). Deletion of *DAL81* significantly reduced white-to-opaque switching, while overexpression of *DAL81* and *GCN4* increased white-to-opaque switching by 5‑10 fold (Fig. S7G). We therefore conclude that Dal81 and Gcn4 are positive regulators of white-to-opaque switching, while Stp2 and Rpn4 are negative regulators of this switch.

We note that several of these factors are also closely linked to the regulation of filamentation in *C. albicans*. When abundant in the extracellular environment, amino acids are imported via amino acid permeases whose expression is controlled by the transcription factor Stp2. As amino acids are metabolized excess N is excreted, thereby raising the environmental pH and inducing filamentation (18, 102, 109). We confirmed that deletion of Stp2 reduces filamentation of cells grown on N peptides, whereas it increases white-to-opaque switching on these substrates (Fig. S7G, and discussed below). In the opposite scenario, amino acid starvation activates the transcription factor Gcn4, which promotes the expression of amino acid biosynthetic genes (103, 110), and Gcn4 also acts to promote white-to-opaque switching (Fig. S7G). We therefore propose that amino acid control over white-opaque switching occurs via Gcn4 and Stp2. During starvation, Gcn4 stimulates white-to-opaque switching, whereas during times of amino acid abundance Stp2 inhibits switching.

**Global analysis of *EFG1*, *HGT4* and *STP2* on *C. albicans* phenotypes**

To further investigate relationships between metabolism, phenotypic switching, and filamentation, we examined *EFG1, HGT4*, and *STP2* deletions for their effects on *C. albicans* when grown on different nutrients. Efg1 is both a key regulator of the switch and modulates the interplay between glycolysis and oxidative metabolism (46), Hgt4 is a high affinity glucose sensor (60, 69), and Stp2 regulates expression of amino acid permease genes and mediates alkalinization of the extracellular environment (18, 102). All three genes displayed strong effects on white-opaque switching frequencies when grown on standard *in vitro* conditions (1-2% glucose and 2% amino acid mixture, Fig. 6E-F, S6-7).

At 37°C, the most notable phenotype of *efg1*∆ white cells was the decrease in both filamentation and overall metabolic fitness compared to wild type cells (Fig. S8A). These mutants also displayed elevated white-to-opaque switching across the majority of C sources tested. With *hgt4*∆ cells, we noted that opaque mutants grown at 37°C displayed increased levels of opaque cell stability when grown on C substrates, but also had markedly reduced fitness compared to wild type cells (Fig. S8B). This mutant also showed increased pseudohyphal growth in opaque cells, which was surprising given its established role in promoting filamentation in white cells (60) and reinforces differences between white and opaque filamentation programs (59, 80, 83). Finally, the *stp2*∆ mutant displayed increased white-to-opaque switching at 25°C on diverse N substrates, but was associated with reduced filamentation (possibly due to decreased alkalinization) and reduced fitness compared to wild type cells (Fig. S8C).

In all three mutants, an increased tendency to form one cell type over the other (either due to increased white-to-opaque switching or increased opaque cell stability) was strongly associated with changes in fitness and filamentation. Importantly, these phenotypic changes extended over a range of nutritional substrates, further establishing that regulation of the white-opaque switch is closely interlinked with both metabolism and filamentation though multiple signaling pathways.

**Metabolic and thermal cues modulate cell type fitness and biofilm formation**

The competitive fitness of white and opaque cells was compared at 37°C (Fig. S9) similar to experiments performed at 25°C (Fig. 9). Whereas opaque cells outcompeted white cells in the presence of glucose-triGly at 25°C, at 37°C white cells were as fit, if not fitter, than opaque cells under all of the tested nutrient conditions (Fig. S9A). The majority of opaque cells switched to the white state when grown in the presence of glucose (irrespective of the N source), whereas a significant fraction of cells in the white state underwent switching to the opaque state when grown with GlcNAc (Fig. S9B). These results again demonstrate how temperature and nutritional cues differentially impact both the fitness of the two cell states, as well as regulate phenotypic switching.

In biofilm assays, white cells formed more robust biofilms than opaque cells at 37°C (Fig. S9C), supporting the idea that the white state is the default cell state at this temperature. In general, biofilm formation of white cells was also significantly more efficient at 37°C than at 25°C (compare Fig. 9C and Fig. S9C). However, conditions that promoted, opaque cell stability and opaque cell filamentation (addition of GlcNAc or triGly) improved biofilm formation at 37°C. We note that these conditions supported biofilm formation by a mixture of white and opaque cells.
