# Supplementary figures and images for: Phenotypic Profiling Reveals that Candida albicans Opaque Cells Represent a Metabolically Specialized Cell State Compared to Default White Cells
Source: mBio. 2016 Nov 22;7(6):e01269-16. doi: 10.1128/mBio.01269-16 (PMC5120136; doi:10.1128/mBio.01269-16)

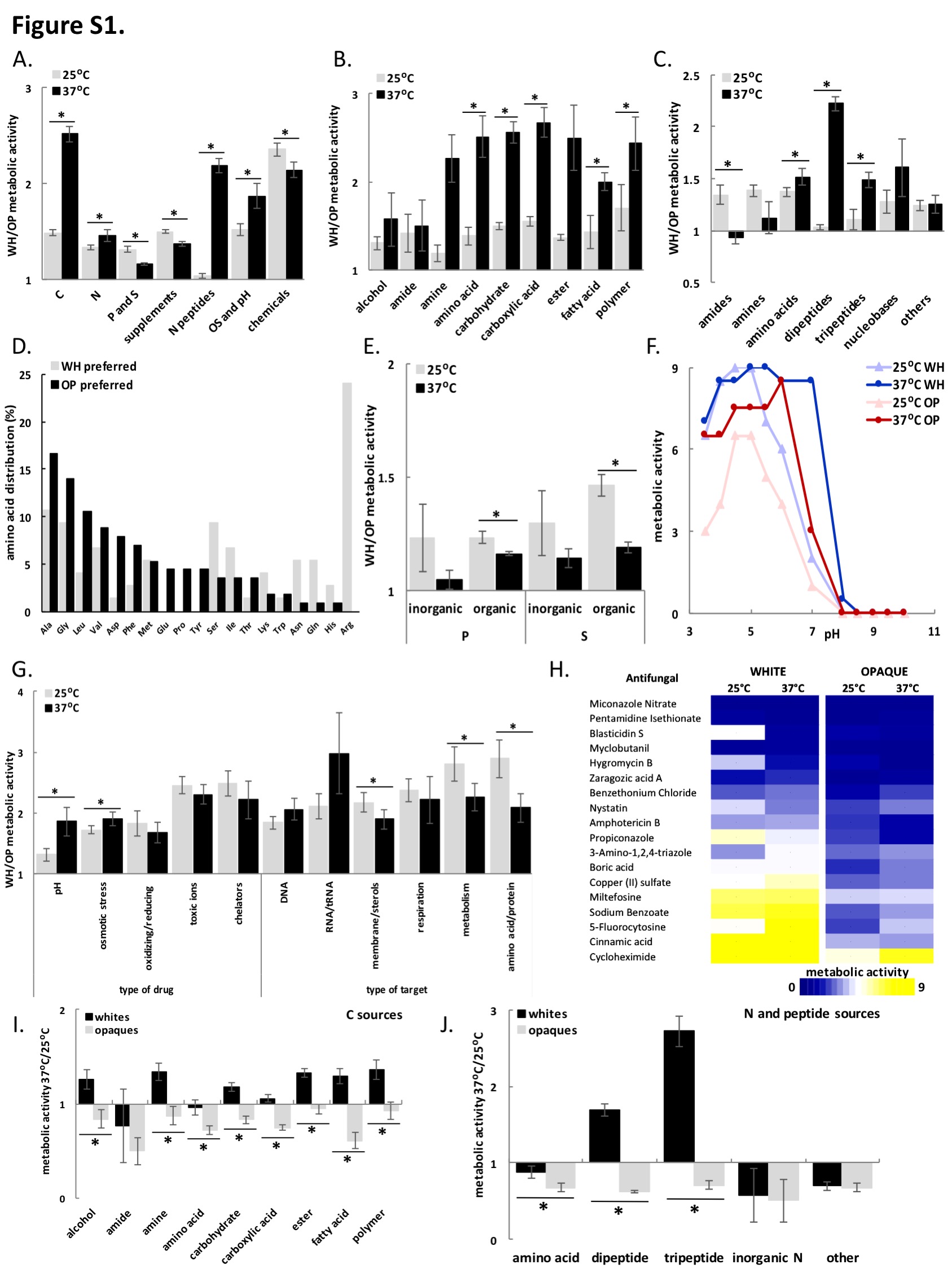

Supplement: Figure S1 — Detailed metabolic analysis across subsets of nutrients and chemicals. (A) Metabolic activity ratios of white cells versus opaque cells (WH/OP) across substrate groups at 25°C and 37°C. (B, C, and E) Averaged WH/OP metabolic activity ratios for subtypes of C sources (B), N and peptide sources (C), and P and S sources (E). (D) Amino acid distribution among peptides preferred by white cells and opaque cells (dipeptides and tripeptides). Cell type preference was calculated based on metabolic activity ratios (white cells preferred WH/OP ratios of ≥1.5, opaque cells preferred OP/WH ratios of ≥1.5). Amino acids were classified based on how often they were present in the sequences of these peptides (shown as a percentage). (F) Metabolic activities of white (blue) and opaque (red) cells grown at different pH levels at 25°C and 37°C. (G and H) White cells and opaque cells have different sensitivities to chemicals and antifungals. (G) Averaged WH/OP metabolic activity ratios shown for different osmolytes and chemicals clustered by the type of drug (left) or mode of action (right). (H) Metabolic activities of white and opaque cells grown on different antifungal agents at 25°C and 37°C. (I and J) Impact of temperature on cell type fitness upon growth on different subsets of C and N substrates. Averaged ratios of 37°C/25°C metabolic activities of white and opaque cells across different subtypes of C sources (I) and N sources (J) (asterisks denote significant differences [P < 0.05] between temperatures [A, B, C, E, G] or between white and opaque cells [I, J]). Download [file mbo006163081sf1.jpg]

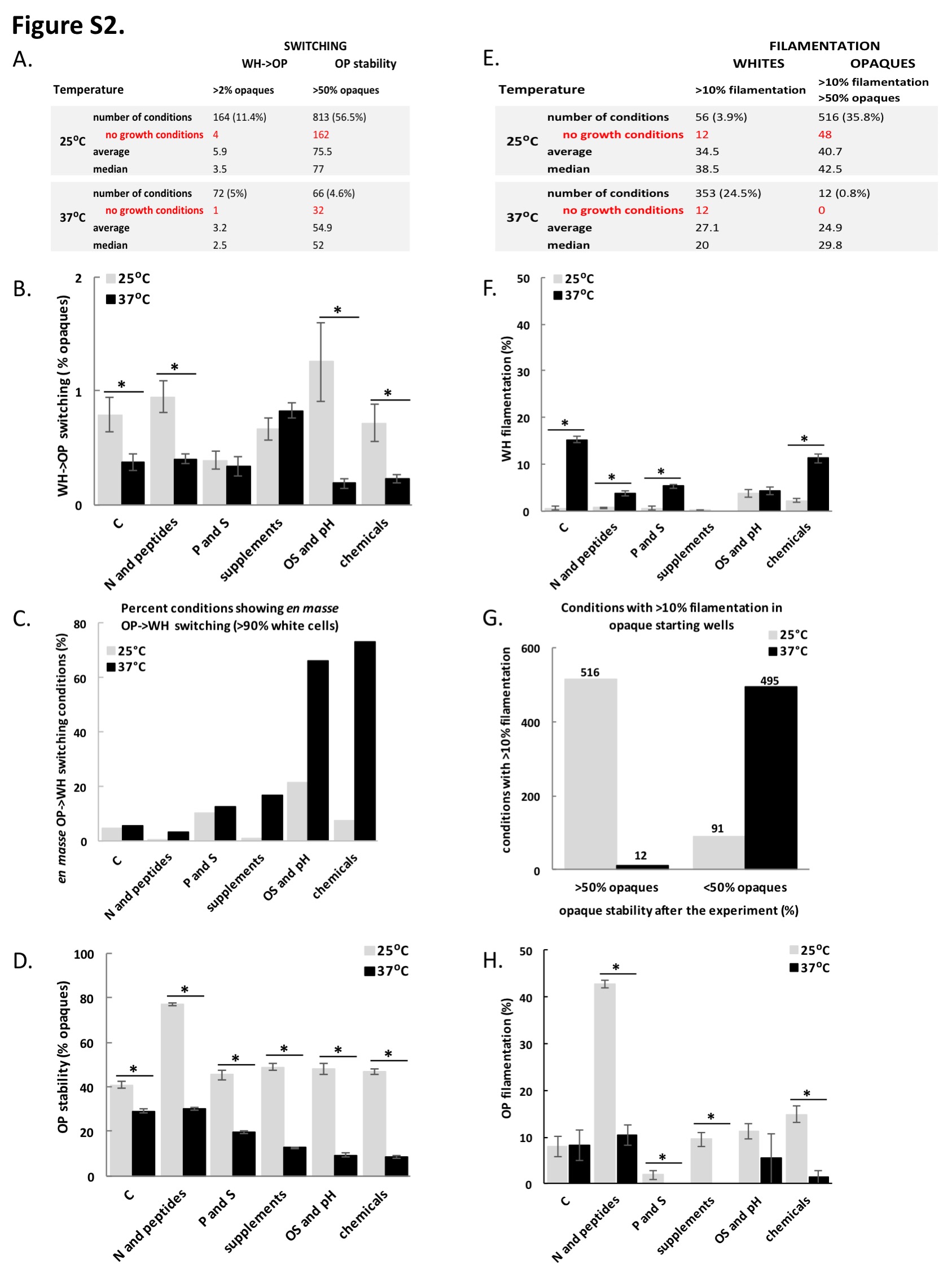

Supplement: Figure S2 — Impact of metabolism on white-opaque switching and filamentation. (A) Summary of the number of conditions inducing WH→OP switching (>2% opaque cells at the end of the experiment) and OP stability (>50% opaque cells at the end of the experiment) at 25°C and 37°C. The numbers of conditions with no growth over the course of the experiment are indicated in red. (B) WH→OP switching levels at 25°C and 37°C averaged across different types of substrates, shown as percent opaque cells in the wells at the end of the experiment. (C) Percentage of conditions inducing en masse OP→WH switching (>90% white cells at the end of the experiment) for each substrate category. (D) Opaque stability levels at 25°C and 37°C averaged across different types of substrates and shown as percent opaque cells in the wells at the end of the experiment. Asterisks denote significant differences (P < 0.05) between the values for cells grown at different temperatures. (E) Summary of the number of conditions inducing white filamentation (>10% of cells at the end of the experiment) and opaque filamentation (>50% opaque cells present and >10% filamentation) at 25°C and 37°C. The numbers of conditions with no growth over the course of the experiment are indicated in red. (F) Filamentation levels at 25°C and 37°C in starting white populations averaged across different types of substrates. (G) Among conditions with >10% filamentation in “opaque starting wells,” those filamenting at 25°C or 37°C were associated with high or low opaque cell stability, respectively. (H) Opaque cell filamentation levels at 25°C and 37°C in “starting opaque populations” averaged across different types of substrates. Only wells that displayed >50% opaque cells at the end of the experiment were included in these statistics. For panels B and D, asterisks denote significant differences (P < 0.05) between the values for cells grown at different temperatures. Download [file mbo006163081sf2.jpg]

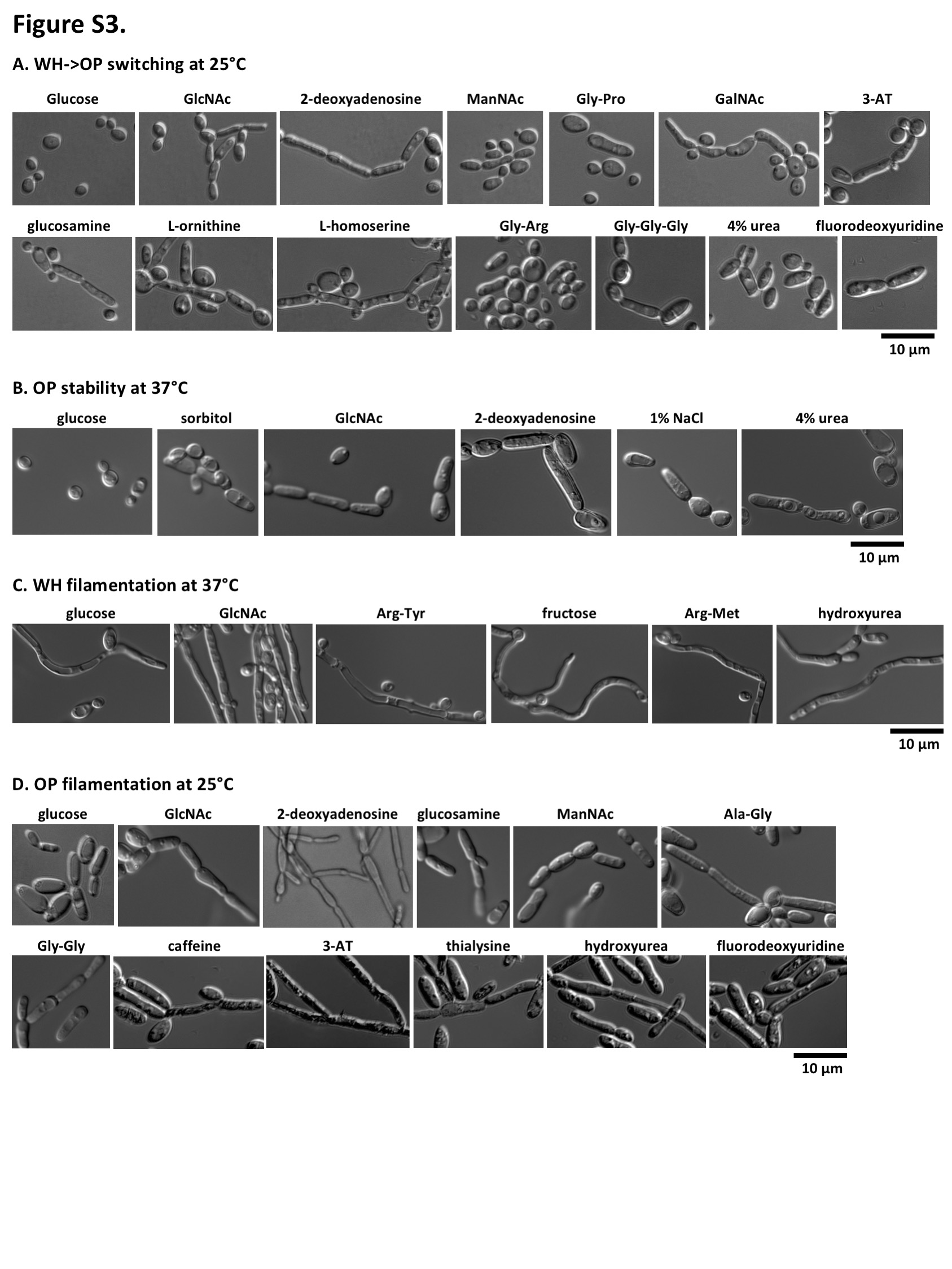

Supplement: Figure S3 — Representative microscopy images of wild-type C. albicans cells displaying white-to-opaque switching at 25°C (A), opaque stability at 37°C (B), white filamentation at 37°C (C), and opaque filamentation at 25°C (D). Download [file mbo006163081sf3.jpg]

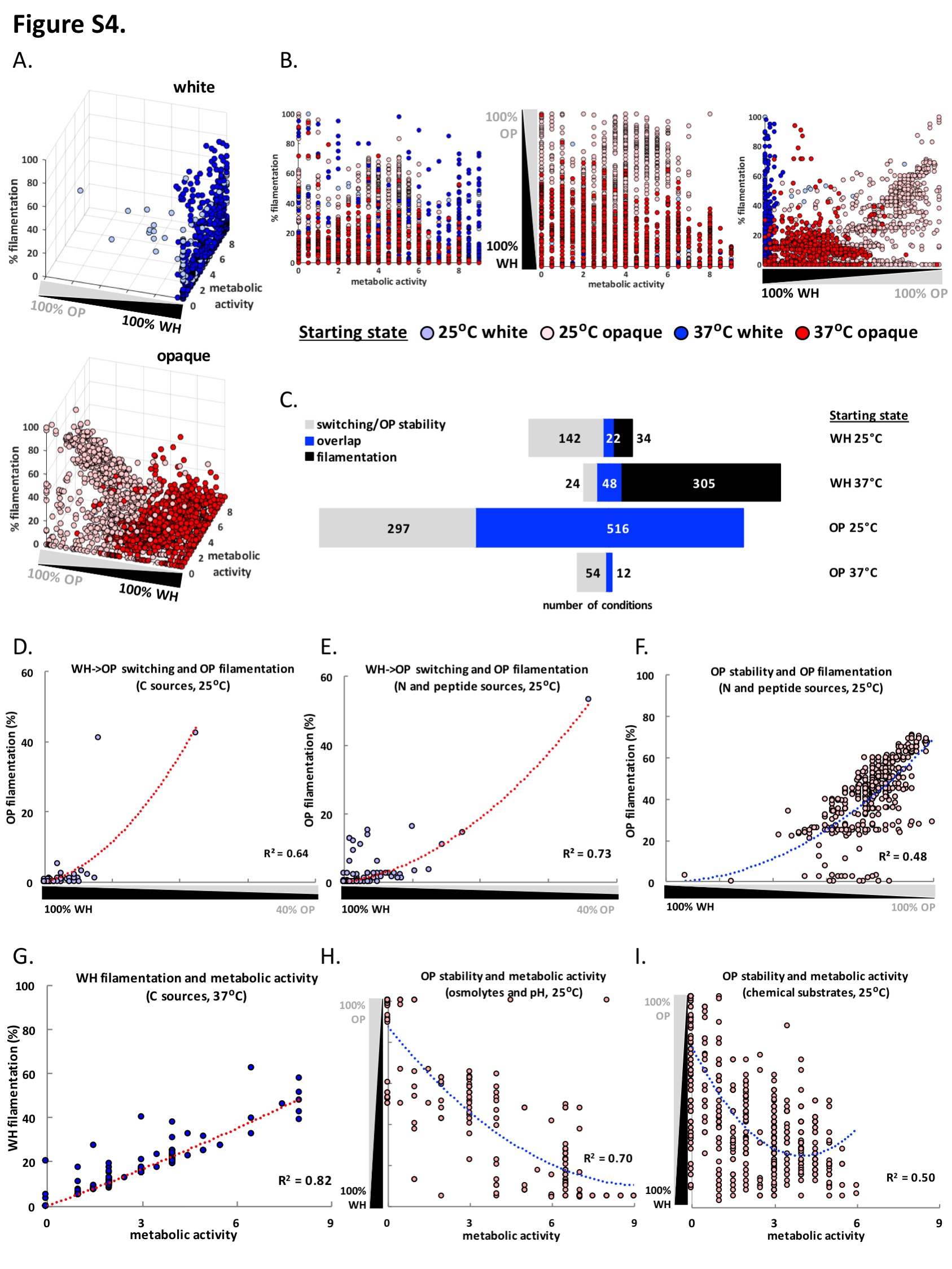

Supplement: Figure S4 — Phenotypic diversity as a function of cell state, filamentation, and temperature. (A) White (left) and opaque (right) wild-type cells grown under 1,440 conditions at 25°C and 37°C plotted in a 3D space defined by fitness (metabolic activity), cell state (percent opaque cells), and degree of filamentation (percent filamentation). (B) No simple correlation defines the relationships between any two parameters of the three phenotypes examined (metabolic activity, white-opaque switching, and filamentation) across all substrates tested. (C) Overlap between conditions that induce switching (>2% opaque cells) and filamentation (>10%) in white cell starting populations or between conditions that stabilize the opaque state (>50% opaque cells) and induce filamentation (>50% opaque cells and >10% filamentation) in opaque starting populations. (D to I) Relationships between different phenotypic parameters (metabolic activity, white-opaque switching, and filamentation). Correlations between WH→OP switching and OP filamentation on C sources at 25°C (D) (PM01 and PM02) and on N and peptide sources at 25°C (E) (PM03 and PM06 to PM08). Note that for these panels, the percent opaque scale goes only to 40%. (F) Correlation between OP stability and filamentation on N and peptide sources at 25°C (PM03 and PM06 to PM08). (G) Correlation between metabolic activity and filamentation of white cells on C source plates at 37°C (PM01 and PM02). Correlations between metabolic activity and opaque stability at 25°C on osmolytes and pH substrates (H) (PM09 and PM10) and chemical substrates (I) (PM21 to PM25). All trend lines were fitted to polynomial regressions. Download [file mbo006163081sf4.jpg]

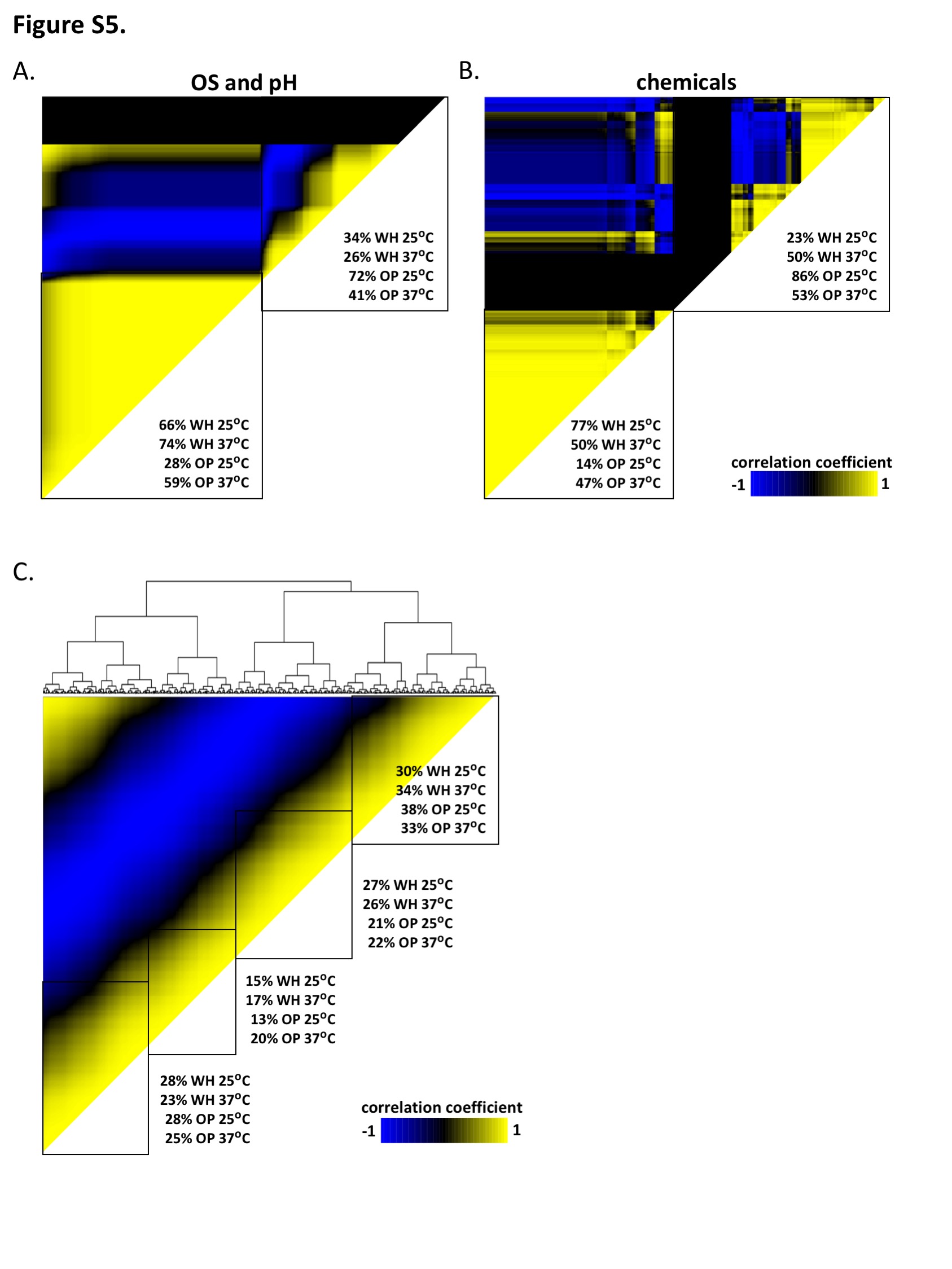

Supplement: Figure S5 — Hierarchical clustering of the OS/pH (PM09 and PM10) and chemical substrates (PM21 to PM25) conditions for the four starting states. Clustering was based on calculating Pearson product moment coefficients (ranging from −1 to 1) between any two conditions. Division of clusters was assigned based on resulting clustergram linkage, and only first- and second-order clusters are shown as a percentage of each starting state. Blocks of black areas represent substrates with a 0 correlation coefficient, and for most cases, these represent wells with no growth. (C) Hierarchical clustering on a uniform set of random parameters for metabolic activity, switching (percent opaque cells), and filamentation (percent) for 250 conditions of each of the four starting states (total of 1,000 conditions). The random phenotypic data set was generated using MATLAB and was used as a control for the clustering analysis. Clustering was based on calculating Pearson product moment coefficients (ranging from −1 to 1) between any two conditions. Division of clusters was assigned based on resulting clustergram linkage, and only first- and second-order clusters are shown as a percentage of each starting state. Clustergram and heat maps for this random data set show the absence of clustering and that the four starting states group uniformly with each other. Download [file mbo006163081sf5.jpg]

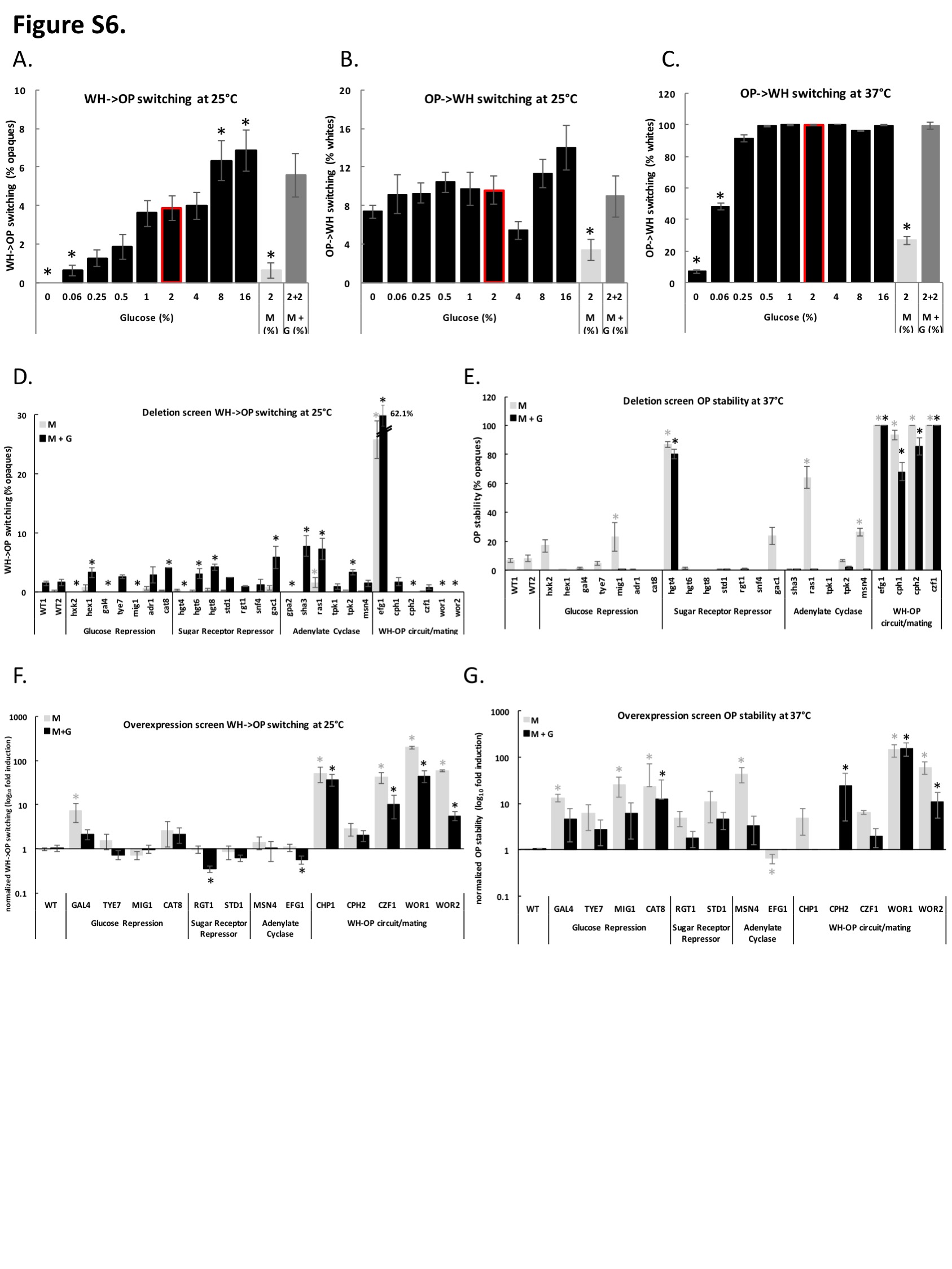

Supplement: Figure S6 — Impact of glucose on phenotypic transitions. Wild-type cells were grown in the presence of increasing glucose concentrations (0 to 16%), 1% mannitol (M), or 1% glucose plus 1% mannitol (M+G), and phenotypic switching rates were assayed on SC plates. Results represent averaged data performed in four to six biological replicates. (A to C) WH→OP switching was monitored at 25°C (A), whereas OP→WH switching was monitored at both 25°C (B) and 37°C (C). Results are shown as percent opaque cells (A) or percent white cells (B and C), and asterisks denote significant differences (P < 0.05) relative to the value for cells grown in 2% glucose condition (marked in red). (D to G) Genetic screens to determine the impact of glucose-related components on white-opaque switching. The impact of gene deletion or overexpression of components of the glucose sensing pathways, the white-opaque transcriptional circuit, or the mating pathway on phenotypic transitions was monitored on SCM and SCM+G media. (D and E) Changes in white-opaque switching (at 25°C) and opaque cell stability (at 37°C) for deletion strains are shown as percent opaque cells. (F and G) Changes in white-to-opaque switching (at 25°C) and opaque cell stability (at 37°C) for doxycycline-inducible overexpressing strains are shown as log10 fold induced changes relative to the values for no-treatment controls (+dox/−dox). Asterisks denote significant differences (P < 0.05) relative to the value for the parental control strain containing an empty vector (D, E, F, G). Download [file mbo006163081sf6.jpg]

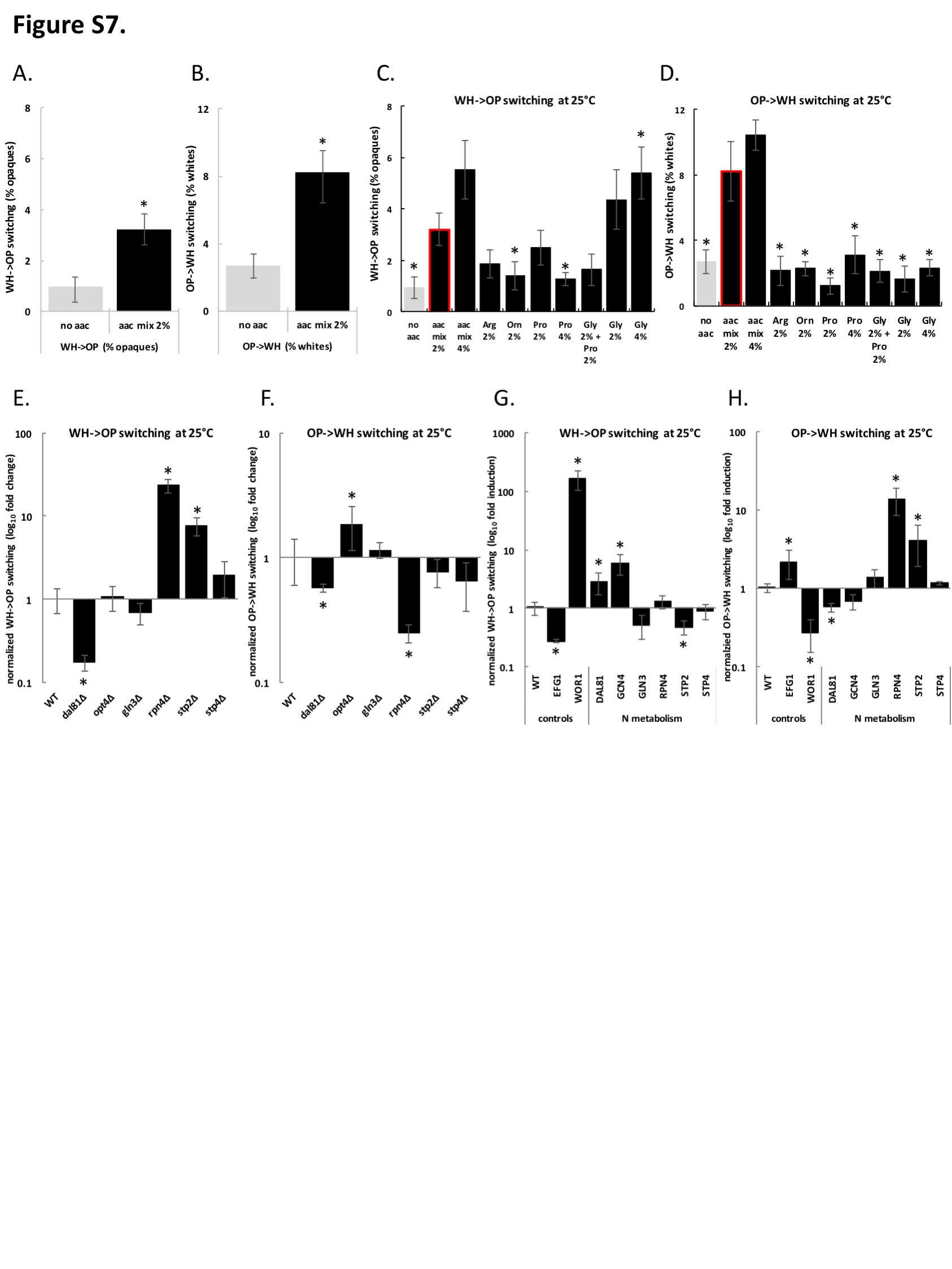

Supplement: Figure S7 — Impact of modulating the amino acid source on white-opaque phenotypic switching. (A and B) Phenotypic switching at 25°C of wild-type C. albicans cells grown either with or without a 2% amino acid mixture. Results represent averaged data from four to six biological replicates. Histograms show percent opaque cells (A) or percent white cells (B) following growth on SCD plates for 7 or 8 days at 25°C (asterisks denote significant differences [P < 0.05] relative to the cells grown without amino acids). (C and D) WH→OP (C) and OP→WH (D) phenotypic switching of wild-type cells grown on different amino acid sources. Histograms show phenotypic switching rates following growth on SCD plates for 7 or 8 days at 25°C (asterisks denote significant differences [P < 0.05] relative to cells grown in the 2% amino acid condition). (E to H) Impact of modulating N metabolism components on phenotypic transitions. Impact of disrupting N metabolism components on white-to-opaque (E) and opaque-to-white switching (F) at 25°C. Histograms show switching rates normalized to the rate of the wild-type control strain (on a log10 axis) and based on percent opaque (E) or white (F) colonies following growth on SCD plates for 7 or 8 days at 25°C. (G and H) Impact of overexpressing N metabolism components on white-to-opaque switching (G) and opaque-to-white switching (H) at 25°C. Histograms show log10 fold induction (+dox/−dox) in phenotypic switching rates following growth on SCD plates for 7 or 8 days at 25°C (asterisks denote significant differences [P < 0.05] compared to the value for the parental control strain [E, F, G, H]). Download [file mbo006163081sf7.jpg]

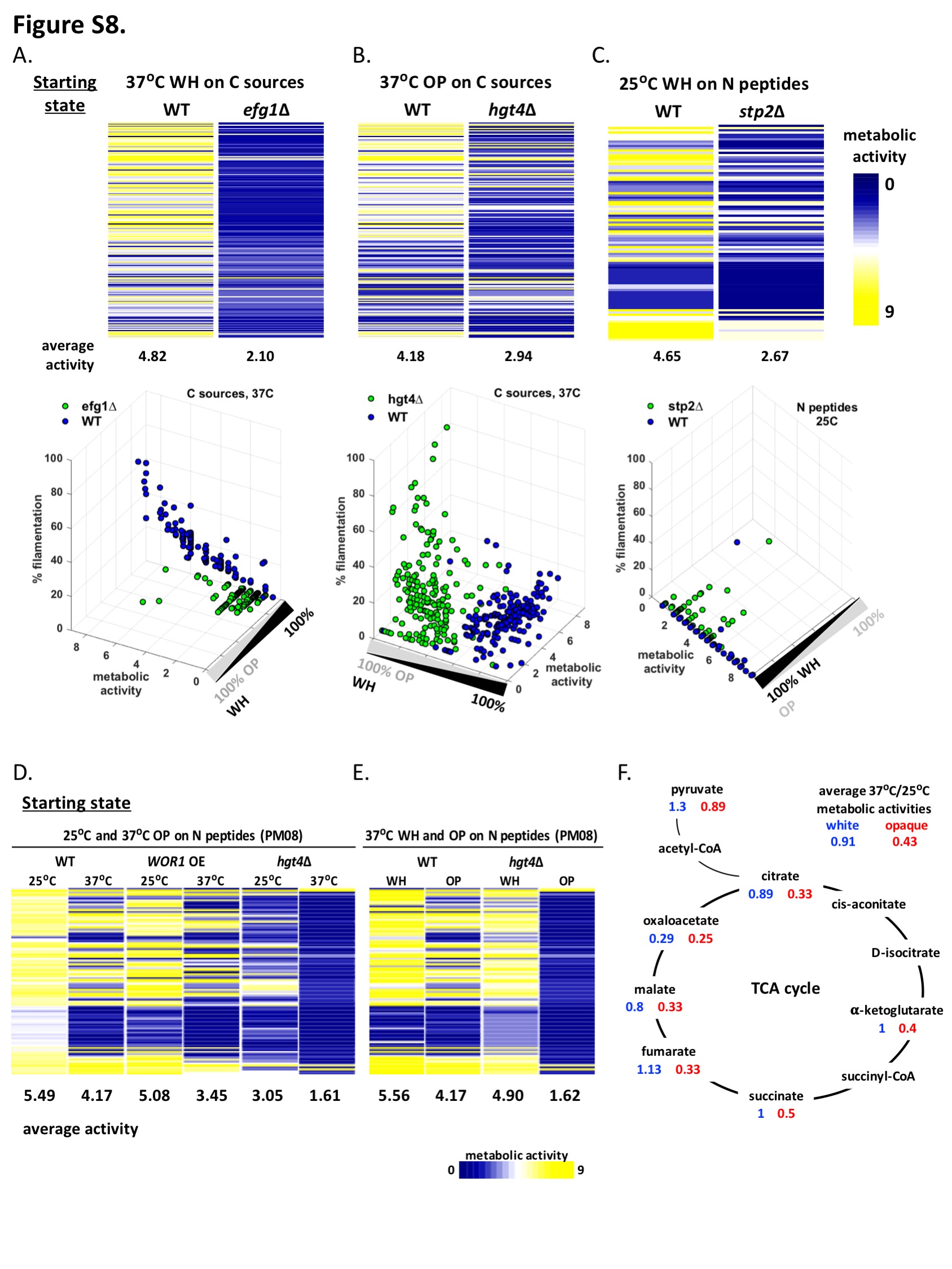

Supplement: Figure S8 — (A to C) Contributions of EFG1, HGT4, and STP2 to the coordinated regulation of metabolism, filamentation, and phenotypic switching in C. albicans. For each analysis, the top panel shows the metabolic activity of strains, and the bottom panel shows the phenotypes of cells compared for metabolic activity, phenotypic switching, and filamentation. (A) Comparison of wild-type and efg1Δ white cells grown on different C substrates (PM01 and PM02) at 37°C. (B) Analysis of wild-type and hgt4Δ opaque cells grown on different C substrates (PM01 and PM02) at 37°C. (C) Analysis of wild-type and stp2Δ white cells grown on different N substrates (PM08) at 25°C. (D to F) Impact of temperature on opaque cell growth at 37°C. (D) Analysis of temperature effects on opaque cell fitness in strains with different opaque cell stability levels. Heat map of metabolic activity of WT, WOR1 OE, and hgt4Δ opaque cells grown at 25°C and 37°C for 24 h on N peptide plates (PM08). (E) Heat map of metabolic activity of WT and hgt4Δ white and opaque cells grown at 37°C for 24 h on N peptide plates (PM08). Metabolic activity is represented on a scale from blue (no growth [metabolic activity value of 0]) to yellow (maximum growth [metabolic activity value of 9]). (F) Schematic representation of the C. albicans TCA cycle, showing the 37°C/25°C metabolic activity ratios on substrates that are part of the central C metabolic pathway. Ratios corresponding to white cells (blue) are significantly higher than those of opaque cells (red), reflecting the decreased functioning of this pathway in opaque cells at 37°C (P < 0.05). Download [file mbo006163081sf8.jpg]

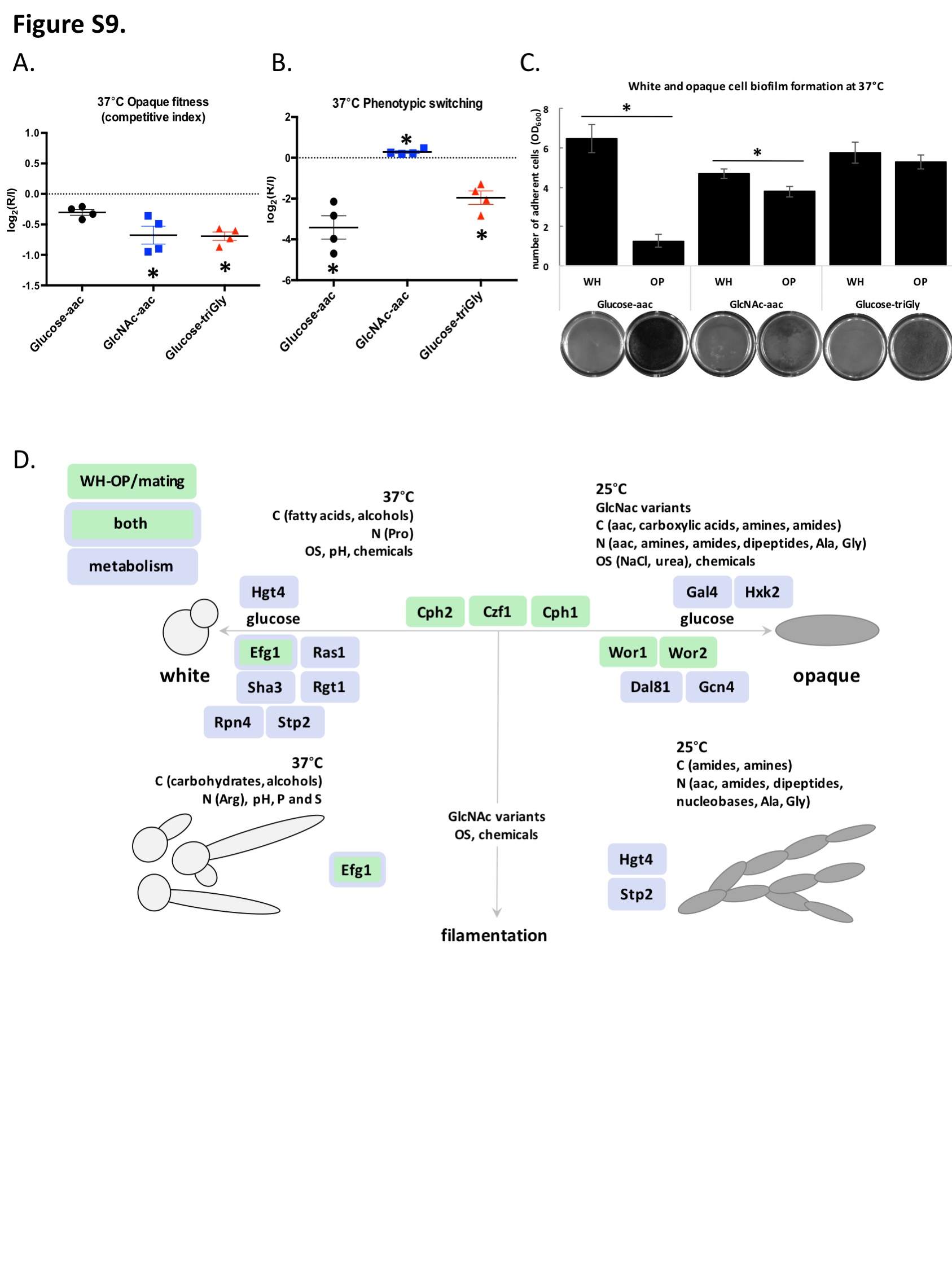

Supplement: Figure S9 — Metabolic rewiring of white and opaque cells impacts fitness and biofilm formation outcomes. (A and B) Growth in a mixed population of wild-type white and opaque cells on different C and N sources at 37°C. Cells were grown on minimal medium with glucose and amino acids, GlcNAc and amino acids, or glucose and triGly without amino acids. Histograms show the opaque cell competitive index and the rate of phenotypic switching calculated as the log2(R/I). R represents the recovered opaque population after 24 h of growth starting from a 1:1 white-opaque mix as the initial population (I). Relative changes were calculated using genetic selection (A) (either white or opaque cells carried a resistance marker) or visual inspection of colony morphologies (B) (by plating cells at the end of the 24-h period). Asterisks denote significant differences (P < 0.05) relative to the values for the initial population. (C) Biofilm formation by white and opaque wild-type cells grown on different C and N sources at 37°C. Cells were grown on Lee’s medium with glucose and amino acids, Lee’s medium with GlcNAc and amino acids, or Lee’s medium with glucose and triGly without amino acids. Histograms show OD600 values following resuspension of adherent cells after 24 h of growth. Images below show representative wells, and asterisks denote significant differences (P < 0.05) between the values for cell types. (D) Differential integration of metabolic and thermal cues by C. albicans white and opaque cells. Schematic summarizing several of the main findings of this study. Formation of both cell types (white/opaque) as well as their specific filamentation programs are favored by distinct cues—they are induced by different metabolic conditions and are wired for growth at different temperatures. Besides known components of the white-opaque circuit, we also highlight novel regulators impacting the switch. Certain substrates have complex roles in modulating phenotypic transitions in more than one directio [file mbo006163081sf9.jpg]
